# MicroRNA-155 Controls *i*NKT Cell Development and Lineage Differentiation by Coordinating Multiple Regulating Pathways

**DOI:** 10.3389/fcell.2020.619220

**Published:** 2021-01-12

**Authors:** Jie Wang, Kai Li, Xilin Zhang, Guihua Li, Tingting Liu, Xiaojun Wu, Stephen L. Brown, Li Zhou, Qing-Sheng Mi

**Affiliations:** ^1^Center for Cutaneous Biology and Immunology Research, Department of Dermatology, Henry Ford Health System, Detroit, MI, United States; ^2^Immunology Research Program, Henry Ford Cancer Institute, Henry Ford Health System, Detroit, MI, United States; ^3^Department of Radiation Oncology, Henry Ford Health System, Detroit, MI, United States

**Keywords:** microRNA-155, *i*NKT cells, innate CD8 T cells, *Jarid2*, *Rictor*, *Tab2*

## Abstract

The development of invariant natural killer T (*i*NKT) cells requires a well-attuned set of transcription factors, but how these factors are regulated and coordinated remains poorly understood. MicroRNA-155 (miR-155) is a key regulator of numerous cellular processes that affects cell development and homeostasis. Here, we found that miR-155 was highly expressed in early *i*NKT cells upon thymic selection, and then its expression is gradually downregulated during *i*NKT cell development. However, the mice with miR-155 germline deletion had normal *i*NKT cell development. To address if downregulated miR-155 is required for *i*NKT cell development, we made a CD4Cre.miR-155 knock-in (KI) mouse model with miR-155 conditional overexpression in the T cell lineage. Upregulated miR-155 led to interruption of *i*NKT cell development, diminished *i*NKT17 and *i*NKT1 cells, augmented *i*NKT2 cells, and these defects were cell intrinsic. Furthermore, defective *i*NKT cells in miR-155KI mice resulted in the secondary innate-like CD8 T cell development. Mechanistically, miR-155 modulated multiple targets and signaling pathways to fine tune *i*NKT cell development. MiR-155 modulated *Jarid2*, a critical component of a histone modification complex, and *Tab2*, the upstream activation kinase complex component of NF-κB, which function additively in *i*NKT development and in promoting balanced *i*NKT1/*i*NKT2 differentiation. In addition, miR-155 also targeted *Rictor*, a signature component of mTORC2 that controls *i*NKT17 differentiation. Taken together, our results indicate that miR-155 serves as a key epigenetic regulator, coordinating multiple signaling pathways and transcriptional programs to precisely regulate *i*NKT cell development and functional lineage, as well as secondary innate CD8 T cell development.

## Introduction

Invariant natural killer T (*i*NKT) cells are innate-like T cells, which possess the properties of both T cells and NK cells, as they coexpress a rearranged T cell receptor (TCR) and several NK cell receptors, including NK1.1. *i*NKT cells are restricted to the non-classical MHC-I-like molecule CD1d and preferentially use an invariant TCR consisting predominantly of the Vα14-Jα18/Vβ8 pair in mice, endowing *i*NKT cells with specificity for self or non-self-glycolipid antigens (Bendelac et al., 2007; Das et al., 2010). Classically, *i*NKT cells undergo four successive developmental stages, including stage 0 (CD24^+^), stage 1 (CD24^–^CD44^lo^), stage 2 (CD44^hi^NK1.1^–^), and stage 3 (CD44^hi^NK1.1^+^). Unlike conventional T cells, *i*NKT cells acquire an effector function and differentiate into *i*NKT1 (PLZF^lo^T-bet^hi^), *i*NKT2 (PLZF^hi^RORγt^–^), and *i*NKT17 (PLZF^int^RORγt^+^) based on transcriptional factors, including T-bet, PLZF, GATA3, and RORγt, before thymic export. Alternatively, *i*NKT1 cells develop through all stages and finally mature in stage 3, which also highly express cytolytic effectors (such as perforin, granzyme B, granzyme A, and FAS ligand) and specific chemokines (such as CCL5) and their receptors. However, *i*NKT2 and *i*NKT17 cells terminate at stage 2. Stage 2 *i*NKT cells have a high proliferation capability, and most *i*NKT cells at this stage emigrate to peripheral organs. Upon antigenic stimulation, *i*NKT1/2/17cells rapidly secrete a broad range of cytokines such as IFN-γ, IL-4, and IL-17, respectively, which mediates functions that link innate and acquired immunity in a broad spectrum of diseases, including infection, cancer, allergy, and autoimmunity (Lee et al., 2013). A core transcription factor network necessary for *i*NKT cell development and functional lineage differentiation has been described. However, how these transcriptional factors are integrated in posttranscriptional and epigenetic programs and what the detailed underlining molecular networks are remained to be explored.

MicroRNAs (miRNAs), a class of 21- to 25-nt single-stranded non-coding small RNAs are emerging as key regulators of numerous cellular processes that affect cell development, homeostasis, and disease development (Bartel, 2004). MiRNAs repress their target genes post-transcriptionally, and one miRNA can potentially target multiple genes depending on the cellular context. Deletion of miRNAs in T cells through targeting Dicer, the ribonuclease III enzyme required for the processing of mature and functional miRNAs, results in severe defects in *i*NKT cell development and function (Zhou et al., 2009). Using different approaches, various groups, including ours, have identified roles for specific microRNAs in *i*NKT cell regulations, such as miR-181a in overall *i*NKT development *via* regulating PI3K signaling and global metabolic fitness (Henao-Mejia et al., 2013); miR-150 in *i*NKT cell maturation through targeting *c-Myb* (Bezman et al., 2011; Zheng et al., 2012); miR183-96-182 control of *i*NKT17 function through targeting *Foxo1* (Wang et al., 2019); and Lethal-7 (let-7) in *i*NKT effector function through regulating PLZF expression (Pobezinsky et al., 2015). Global miRNA expression patterns for human and mouse tissues show that miR-155 is prominently expressed in many hematopoietic cell types (Landgraf et al., 2007) and upregulation of miR-155 has been shown to be a consistent feature of the mammalian inflammatory response (Teng et al., 2008; Vigorito et al., 2013). By using genetically engineered mouse models of miR-155, and through identification of endogenous targets of miR-155, a wide spectrum of previous studies have identified the multiple roles of miR-155 in regulating varieties of immune cell differentiation and immune responses, including B cells, CD8 T cells, and CD4 T cells, as well as for T helper type 1 (Th1), Th2, Th17, and regulatory T cells (Seddiki et al., 2014). In addition, some studies suggest that miR-155 plays a role in *i*NKT cell development and function (Burocchi et al., 2015; Frias et al., 2018). However, the detailed regulatory role of miR-155 in *i*NKT cell functional lineage differentiation and the detailed molecular mechanisms involved remain largely unresolved.

In the present study, we confirmed the dynamic changes of miR-155 expression during thymic *i*NKT cell development and differentiation. To explore the potential role of miR-155 in *i*NKT cell development and differentiation, two distinct but complementary approaches were used. Germline miR-155 deletion in mice did not significantly interrupt *i*NKT cell development, which is largely consistent with previous report (Frias et al., 2018). However, T cell-specific overexpression of miR-155 resulted in defective *i*NKT cell development and differentiation, including compromised *i*NKT1 and *i*NKT17 cell differentiation, and in augmented IL-4-producing *i*NKT2 cell differentiation with promotion of secondary thymic innate CD8 T cell development. Mechanistically, miR-155 controlled *i*NKT cell development, suppressed *i*NKT1, and promoted *i*NKT2 differentiation with secondary enhancement of innate CD8 T cell development through targeting *Jarid2* and *Tab2*; whereas miR-155 inhibited *i*NKT17 differentiation and *i*NKT survival through targeting *Rictor*. The multilayer regulation of nuclear factor kappa B (NF-κB) by miR-155 may be the cause of the self-limiting expression of miR-155 during *i*NKT thymic development. Therefore, the dynamic regulation of miR-155 expression is essential for normal *i*NKT cell thymic development, differentiation, and homeostasis.

## Materials and Methods

### Mice

Conventional miR-155 knockout (miR-155KO) mice in a C57BL/6 background and wild-type (WT) C57BL/6 mice were purchased from The Jackson Laboratory (Bar Harbor, ME, United States). Mice carrying the Rosa26 miR-155 knock-in (KI) allele (Jackson Laboratory) were bred with CD4Cre transgenic mice (Jackson Laboratory) to obtain mice expressing CD4Cre with one or two copies of the miR-155 KI allele. Mice with two copies of miR-155 KI allele, miR-155KI mice (CD4Cre^+^miR-155KI, miR-155KI), and the littermate CD4Cre^–^miR-155KI [wild-type (WT)] mice served as controls. Mice were 6- to 10-week-old, gender-matched, and housed in a specific pathogen-free barrier unit. Handling of mice and experimental procedures were in accordance with requirements of the Institutional Animal Care and Use Committee.

### Flow Cytometry

Single-cell suspensions were washed twice with staining buffer (PBS, 2% FBS) and incubated with Fc block (clone 2.4G2). Cells (2 × 10^6^ per staining well) were stained with antimouse PBS57-loaded CD1d-tetramer and PBS57-unloaded CD1d-tetramer (provided by the NIH Tetramer Core Facility). The following conjugated mAbs were used: TCRβ (H57-597), CD24 (30-F1), CD44 (IM7), CD122 (5H4), CD69 (H1.2F3), NK1.1 (PK136), IL17RB (MUNC33), Slamf1 (TC15-12F12.2), Slamf6 (330-AJ), PLZF (9E12), RORγt (B2D), T-bet (eBio17B7), GATA3 (TWAJ), CD1d (1B1), IL-4 (11B11), IFN-γ (XMG1.2), IL-17a (eBio17B7), CD45.1 (A20), CD45.2 (104), CD4 (GK1.5), CD8 (53-6.7), CXCR3 (CXCR3-173), and Eomes (Dam11mag). All mAbs were purchased from eBioscience, Biolegend, or TOBON. Apoptosis assays were carried out by staining with Annexin V (eBioscience), according to manufacturer’s instructions. Cell surface staining was performed with staining buffer. Intranuclear staining for GZMA, PLZF, T-bet, RORγt, Bcl-2, and Ki-67 was performed with eBioscience Fixation/permeabilization buffer. The flow cytometry assay was performed through BD FACSCelesta, and data were analyzed using FlowJo V10.2 software. Gating strategy: after gating on lymphocyte, doublets were excluded by using forward scatter (FSC) and side scatter (SSC), mouse *i*NKT cells were then identified as TCRβ^+^ CD1d-tetramer^+^ (Supplementary Figures 1A,B).

### *In vitro* Functional Assays

Thymocytes and spleen cells total 4 × 10^6^ from mice were cultured in RPMI-1640 Medium (Sigma, United States) supplemented with 10% FBS, 100 units/ml penicillin, 100 units/ml streptomycin, 25 mM HEPES, 1 mM sodium pyruvate, 1 × non-essential amino acids, 2 mM L-glutamine, and 50 μM 2-mercaptoethanol, in the presence of phorbol 12-myristate 13-acetate (PMA) (50 ng/ml) and ionomycin (1 μM) for a total of 4 hours at 37°C. Brefeldin A was then added in the last 2.5 h at a final concentration of 1 μM. Anti-IFN-γ, anti-IL-4, and anti-IL-17 were detected by intracellular staining and flow cytometry.

### Bone Marrow Chimera Transfer Experiments

To generate bone marrow chimeras, 7- to 8-week-old B6.SJL-recipient mice (CD45.1^+^CD45.2^+^) were lethally irradiated with 900 rad. Donor bone marrows were harvested from age- and sex-matched SJL (CD45.1^+^) mice and miR-155KI mice (CD45.2^+^) by flushing bones with a syringe containing sterile basal tissue culture medium (RPMI 1640 with 10% FBS). After erythrocyte lysis, mature T cells (CD3^+^) were depleted from bone marrow from each donor mouse by biotin-conjugated antimouse CD3 (BD Biosciences, clone17A2) mAbs and antibiotin magnetic beads (BD Biosciences) using Magni Sort^TM^. Over 90% of mature T cell depletion was confirmed by flow cytometry. CD45.1^+^ SJL and CD45.2^+^ miR-155KI or WT control bone marrows were mixed at a 1:1 ratio, and total of 10 × 10^6^ cells per mouse (in a volume of 100 μl) were then injected into the irradiated recipient mice by tail vein injection. The chimeras were analyzed 8 weeks after reconstitution.

### *i*NKT Cells Enrichment and Sorting

Total thymocytes and spleen cells were first prepared at a concentration of 1 × 10^7^/100 μl in staing buffer (PBS, 2% FBS) stained with anti-CD8 biotin Ab (TONBO, clone 53-6.6) for thymocytes or anti-CD8 biotin Ab and anti-B220 biotin Ab (eBioscience, clone RA3-6B2) for spleen cells. CD8^+^ T cells and B220^+^ cells were then depleted with antibiotin beads (MagniSort^TM^) using auto-MACS (Miltenyi Biotec). Negatively selected cells were then stained with anti-TCR-β, anti-CD1d tetramers, anti-NK1.1, and anti-CD44 Abs. Total or different stages of *i*NKT cells were then sorted by BD FACS Aria II.

### RT-qPCR Analysis

*i*NKT cells from WT controls and miR-155KI mice were sorted, and total RNA was extracted by using the EXIQON isolation. RNA was reverse transcribed using the PrimeScript^TM^ RT reagent kit, and RT-qPCR was performed on an Applied Biosystems 7900 Real-Time PCR system with the following primer pairs: *Jarid2* (Forward: 5′-GACACCAAACCCAATCACCAC-3′ and Reverse: 5′-GTTCAACCTGCCACTGACCTT-3′); *Rictor* (Forward: 5′-TGAGTACCGTTCTTCTCAC-3′ and Reverse: 5′-TGCAATGGAGGG CGCTTTA-3′); *Tab2* (Forward: 5′-GGATAGAATAAGCGAAGCCCGGAA-3′ and Reverse: 5′-CTC TTTGAAGCCGTTCCATCCT-3′). Expression levels of miR-155 were normalized with snoRNU202, and expression levels of *Jarid2*, *Rictor*, and *Tab2* were normalized with L32. Fold change was calculated using the 2^−ΔΔ*CT*^ method.

### RNA-Seq

RNA concentration was determined using the Qubit RNA broad range assay in the Qubit Fluorometer (Invitrogen), and RNA integrity was determined using the Eukaryote Total RNA. Nano Series II ChIP on a 2100 Bioanalyzer (Agilent). Three independent biological replicates were pooled for RNA-seq. RNA-seq libraries were prepared using 4 μg of total RNA with the TruSeq RNA sample prep kit following manufacturer’s protocol (Illumina).

### Western Blot and ELISA

Cell lysates were prepared by lysis in RRAP buffer containing protease inhibitor and Western blots were performed using standard methods. Image J^1^ was used to quantify protein expression levels from digital images of Western blots. IL-4 protein concentration in mouse blood serum was measured using the IL-4 mouse ELISA Ready-Set-Go! Kit (eBioscience) following the manufacturer’s instructions.

### Statistical Significance

GraphPad Prism 8.0 was used for statistical analysis (unpaired, two-tailed, *t*-test with 95% confidence interval). A *P*-value < 0.05 was considered statistically significant. The processing of RNA-Seq data was performed with R version 3.6.0^2^.

## Results

### Dynastic Expression of miR-155 During *i*NKT Cell Development

**FIGURE 1:**
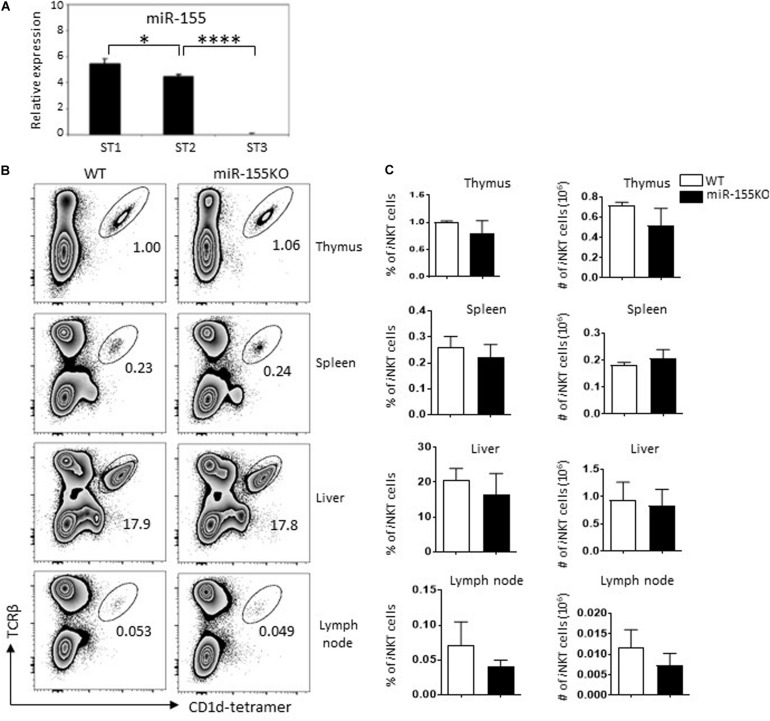
Dynastic expression of miR-155 during *i*NKT cell development. **(A)** Expression of miR-155 in different developmental stages of thymic *i*NKT cells from C57BL/6 mice. Total RNA was obtained from different stages of WT *i*NKT cells, and miR-155 expression was measured by RT-qPCR (snoRNU202 as quantitative control for normalization, three mice for each group). **(B)** Representative flow cytometry plots showing *i*NKT cells (TCRβ^+^ CD1d-tetramer^+^) in thymus, spleen, lymph nodes, and liver of miR-155KO mice and WT controls. Numbers adjacent to outlined areas indicate percentage of indicated populations. **(C)** Bar graphs showing frequencies (left) and the absolute numbers (right) of *i*NKT cells in indicated organs of miR-155KO and WT controls. Data are from two independent experiments, 4–6 mice for each group, data were analyzed by unpaired *t*-test. *P < 0.05, ****P < 0.0001.

To gain insight into the role of miR-155 in the development of *i*NKT cells, we first tested miR-155 expression in sorted thymic *i*NKT cells that were at different developmental stages. Thymocytes from normal C57BL/6 mice were stained with Abs against TCR-β, CD1d-tetramer, CD44, and NK1.1 to identify and sort stage 1 (CD44^lo^NK1.1^–^), stage 2 (CD44^hi^NK1.1^–^), and stage 3 (CD44^hi^NK1.1^+^) *i*NKT cells. MiR-155 expression was then detected by TaqMan real-time PCR. MiR-155 was detected in all developmental stages of *i*NKT cells, and expression was dramatically decreased during *i*NKT cell development, almost to the lowest level at the terminal developmental stage 3 (Figure 1A). These findings indicated that miR-155 is expressed in a stage-specific pattern in *i*NKT cells and that miR-155 expression is significantly repressed in mature *i*NKT cells.

### MiR-155 Deletion Does Not Affect *i*NKT Cell Development

To assess the role of miR-155 in *i*NKT cell development, we performed a loss-of-function study using conventional miR-155 knockout (miR-155KO) mice. Nevertheless, we did not observe a significant *i*NKT cell developmental defect in thymus and peripheral lymph organs in mutant mice. The frequencies and absolute numbers of *i*NKT cells in thymus, spleen, lymph nodes, and liver peripheral organs were comparable between miR-155KO and WT mice (Figures 1B,C). *i*NKT cells lacking miR-155 also showed a normal developmental stage (Supplementary Figure 1C), which was consistent with a previous study (Frias et al., 2018), suggesting that early *i*NKT cell development is not critically dependent on miR-155 expression. Given that miRNA does not exhibit transcriptional activity itself, it acts as a transcriptional inhibitor during the regulation of cellular development. It is possible that the relieved inhibition of target(s) of interested from miR-155 deletion cells in the earliest stages of *i*NKT cells may not be enough for an obvious interruption of *i*NKT cell development.

### MiR-155 Overexpression Interrupts *i*NKT Cell Development

The expression of miR-155 was gradually downregulated during *i*NKT cell differentiation (Figure 1A). To gain further insight into the dynamic regulation of miR-155 during *i*NKT cell development, a gain-of-function gene targeting approach was used. To generate mice with T cell-specific overexpression of miR-155, mice carrying the Rosa26 miR-155 KI allele were bred with CD4Cre transgenic mice to obtain mice expressing CD4Cre with one or two copies of the miR-155 KI allele. Mice with two copies of the miR-155 KI allele, miR-155KI (CD4Cre^+^miR-155KI, miR-155KI), and WT littermate control (CD4Cre^–^miR-155KI, WT) were used in experiments without specific indication (Supplementary Figures 2A–C). To evaluate the magnitude of miR-155 upregulation, both thymus and spleen TCRβ + T cells were sorted, and miR-155 expression was detected and compared with miR-155 KI and littermate WT mice with RT-PCR. As shown in Supplementary Figure 2C, substantial upregulation of miR-155 was observed at about six and three times of WT control T cells in thymus and spleen, respectively.

**FIGURE 2:**
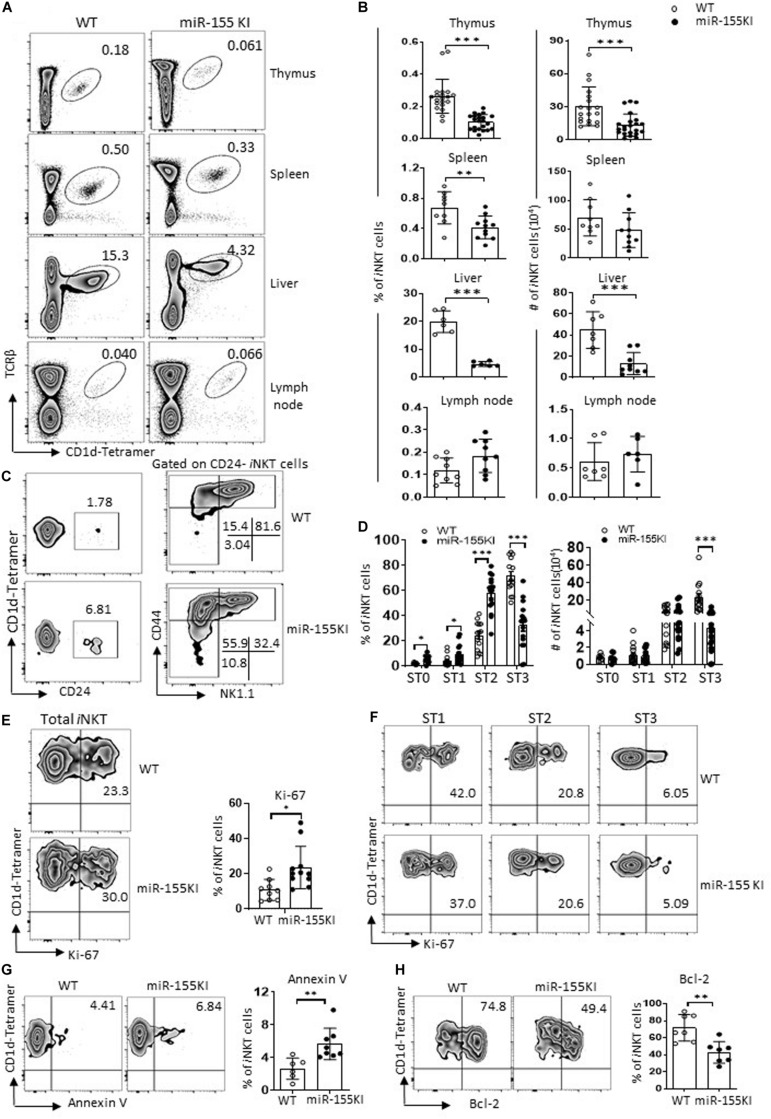
MiR-155 overexpression interrupts *i*NKT cell development. **(A)** Representative flow cytometry plots depicting *i*NKT cells in thymus, spleen, liver, and lymph nodes of WT controls and miR-155KI mice. Numbers adjacent to outlined areas indicate percentage of indicated populations. **(B)** Bar graphs showing frequencies (left) and absolute numbers (right) of *i*NKT cells in indicated organs of miR-155KI and WT controls. **(C)** Representative flow cytometry plots depicting expression of CD24, CD44, and NK1.1 on *i*NKT cells: stage 0 (ST0, CD24^+^), stage 1 (ST1, CD24^–^ CD44^lo^NK1.1^–^), stage 2 (ST2, CD44^hi^NK1.1^–^), and stage 3 (ST3, CD44^hi^NK1.1^+^) in WT controls and miR-155KI mice. **(D)** Bar graphs showing frequencies (left) and absolute numbers (right) of *i*NKT cells in different developmental stages (ST0, ST1, ST2, and ST3) of miR-155KI and WT controls. **(E)** Representative flow cytometry plots depicting expression of Ki-67 in thymic *i*NKT cells (left). Bar graph showing frequency of Ki-67^+^ *i*NKT cells from miR-155KI and WT controls (right). **(F)** Representative flow cytometry plots depicting expression of Ki-67 in different stages (ST1, ST2, and ST3) of *i*NKT cells from miR-155KI and WT controls. **(G,H)** Representative flow cytometry plots depicting expression of Annexin V **(G)** and Bcl-2 **(H)** in thymus *i*NKT cells (left). Bar graphs showing frequency of Annexin V^+^ *i*NKT cells and Bcl-2^+^ *i*NKT cells from miR-155KI and WT controls (right). Data are from at least three independent experiments. Each mouse is represented as one dot. Data were analyzed by unpaired *t*-test. *P < 0.05, **P < 0.01, ***P < 0.001.

To assess the role of miR-155 overexpression in *i*NKT cell development, we first examined the frequency and number of *i*NKT cells in different immune organs including thymus, spleen, liver, and lymph nodes of cells from 6- to 8-week-old miR-155KI and WT control mice by flow cytometry. As shown in Figures 2A,B, miR-155KI mice exhibited decreased frequencies and absolute numbers of *i*NKT cells compared with WT mice in thymus, spleen, and liver but not in lymph node. Cell surface markers distinguishing the stages of thymic *i*NKT cell development are well characterized. The frequencies of stages 0, 1, and 2 *i*NKT cells were relatively higher in miR-155KI mice and were accompanied with a reduced frequency of stage 3 *i*NKT cells. However, the absolute numbers of stages 0, 1, and 2 cells in miR-155KI mice were comparable with those in WT mice, whereas stage 3 *i*NKT cells were dramatically reduced in miR-155KI mice (Figures 2C,D). These data indicate that overexpressing miR-155 impaired *i*NKT cell development, especially for terminal maturation stage 3 (or *i*NKT1) cells.

To test whether cell homeostasis contributes to *i*NKT cell developmental defects in miR-155KI mice, we examined the rate of Ki-67 expression to measure *i*NKT cell proliferation capability. Compared with WT controls, miR-155KI *i*NKT cells exhibited a higher proliferating status (Figure 2E). A previous study indicated that stages 1/2 *i*NKT cells proliferate briskly, while stage 3 mature *i*NKT cells are relatively quiescent (Gapin, 2016). To dissect whether increased Ki-67 expression in overall *i*NKT cells from miR-155KI mice is merely reflection of their defective maturation phenotype, we then further analyzed Ki-67 expression in different developmental stages. As shown in Figure 2F, the expression of Ki-67 was comparable within the same stages of *i*NKT cells in miR-155KI and WT mice. These results indicate the largely dispensable role of miR-155 in the proliferation of *i*NKT cells. However, the staining of fresh thymocytes with the apoptotic marker Annexin V revealed significantly higher levels of apoptotic *i*NKT cells in miR-155KI mice (Figure 2G). Consistently, we found the antiapoptosis marker Bcl-2 was significantly reduced in miR-155KI *i*NKT cells compared with WT (Figure 2H). Taken together, these data indicate that miR-155 overexpression impairs survival of *i*NKT cells, likely contributing to the defect of the overall *i*NKT cell development.

### MiR-155 Overexpression Interrupts *i*NKT1 Cell Differentiation

**FIGURE 3:**
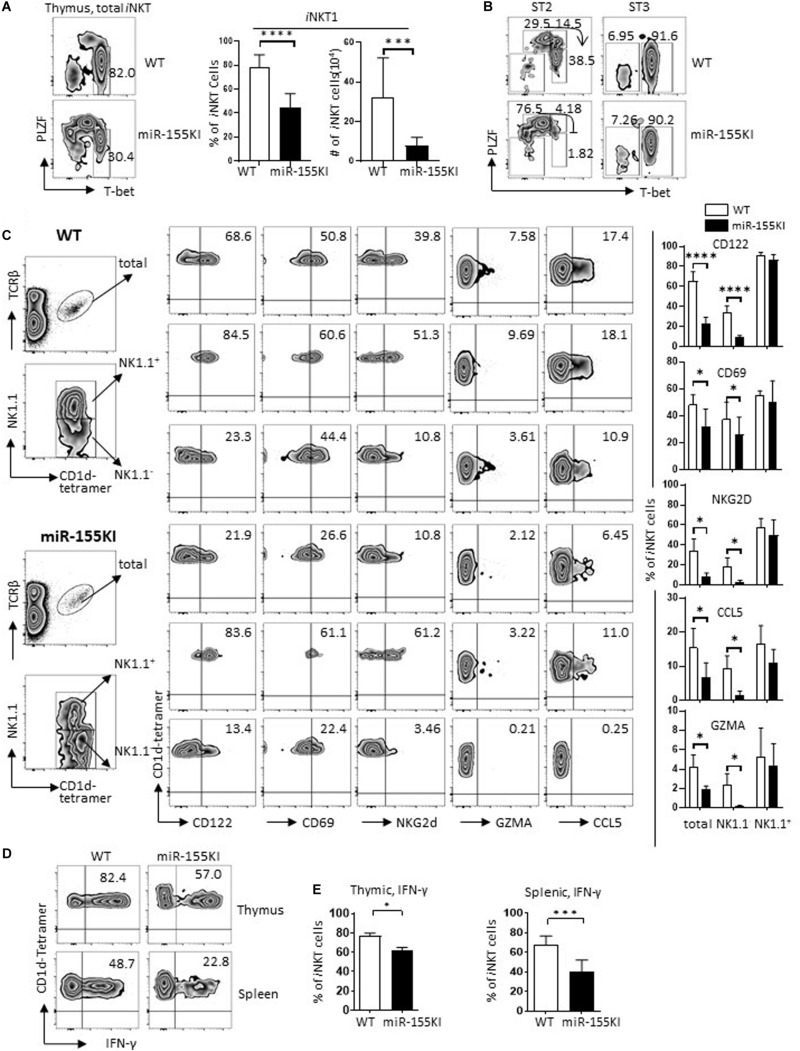
miR-155 overexpression interrupts *i*NKT1 cell differentiation. **(A)** Representative flow cytometry plots depicting *i*NKT1 (PLZF^lo^Tbet^+^) gated on total thymic *i*NKT cells. Bar graphs showing frequency (left) and absolute number (right) of *i*NKT1 cells from miR-155KI and WT controls. **(B)** Representative flow cytometry plots depicting *i*NKT1 (PLZF^lo^Tbet^+^) gated on stage 2 (ST2) and stage 3 (ST3) *i*NKT cells from miR-155KI mice and WT controls. **(C)** Representative flow cytometric plots depicting CD122, CD69, NKG2D, GZMA, and CCL5 expression in total thymic *i*NKT cells (top panel), NK1.1^+^ *i*NKT cells (middle panel), and NK1.1-*i*NKT cells (bottom panel) from miR-155KI mice (bottom) and WT controls (top). Bar graphs showing frequencies of CD122, CD69, NKG2D, GZMA, and CCL5 expression in indicated *i*NKT subsets from miR-155KI mice and WT controls. **(D)** Representative flow cytometry plots depicting IFN-γ production in thymic *i*NKT cells (top) and splenic *i*NKT cells (bottom) from miR-155KI mice and WT controls. **(E)** Bar graphs showing frequency of IFN-γ producing *i*NKT cells in thymus (left) and spleen (right) from miR-155KI and WT controls. Data are from at least five independent experiments. Data were analyzed by unpaired *t*-test. *P < 0.05, **P < 0.01, ***P < 0.001, ****P < 0.001.

*i*NKT cells at different stages are functionally distinct, where the stage 2 *i*NKT pool contains well-differentiated *i*NKT2/17, and immature pre-*i*NKT1 cells, while stage 3 pools contain largely terminally differentiated *i*NKT1 cells (Lee et al., 2013). Considering that stage 3 *i*NKT cells were severely reduced in miR-155KI mice (Figures 2C,D), we therefore analyzed the *i*NKT1 cell signature T-bet (*Tbx21*). Normally, T-bet is initially expressed in stage 1/2 and is maximally expressed in stage 3, while PLZF is gradually downregulated along this developmental process. Here, we found that miR-155KI *i*NKT cells failed to upregulate T-bet at stage 2, resulting in less PLZF^hi/int^ T-bet^+^ *i*NKT cells from stage 2 progressing to stage 3 (Figures 3A,B). Therefore, stage 2 *i*NKT cells from miR-155KI mice contained a smaller proportion of immature *i*NKT1 cells compared with WT mice (Figure 3B). Consistent with this notion, we found dramatically decreased numbers of other *i*NKT maturation markers, including CD122 (*Il2rb*), CD69 (*Cd69*), and NKG2D (*Klrk1*) in miR-155KI thymic *i*NKT cells more specifically, in the NK1.1^–^ counterpart (Figure 3C). In reflection of the thymic *i*NKT cell maturation defect, peripheral *i*NKT cells in miR-155KI mice also showed a relatively immature phenotype as judged by reduced NK1.1 expression (Supplementary Figure 3). The maturation defective phenotype observed in miR-155KI *i*NKT prompted us to further analyze the expression of *i*NKT cell cytotoxic markers, which were raised from *i*NKT1 cells. We found that the expression of CCL5 (*Ccl5*) and GZMA (*Gzma*) were significantly reduced in the miR-155KI *i*NKT NK1.1^–^ counterpart, suggesting the downregulated cytotoxic capability of *i*NKT cells from miR-155KI mice (Figure 3C).

To characterize the function of *i*NKT cells, total thymocytes and splenocytes from miR-155KI and WT mice were stimulated with PMA/Ionomycin for 4 hours *in vitro*. Consistent with their differentiation phenotype, IFN-γ production was significantly reduced in both thymic and splenic *i*NKT cells from miR-155KI mice (Figures 3D,E). Overall, *i*NKT cells with miR-155 overexpression showed remarkably suppressed *i*NKT1 cell differentiation and maturation. Therefore, the downregulation of miR-155 during thymic *i*NKT cell development is essential for *i*NKT1 cell differentiation and maturation.

### MiR-155 Overexpression Promotes *i*NKT2 Cell Differentiation

**FIGURE 4:**
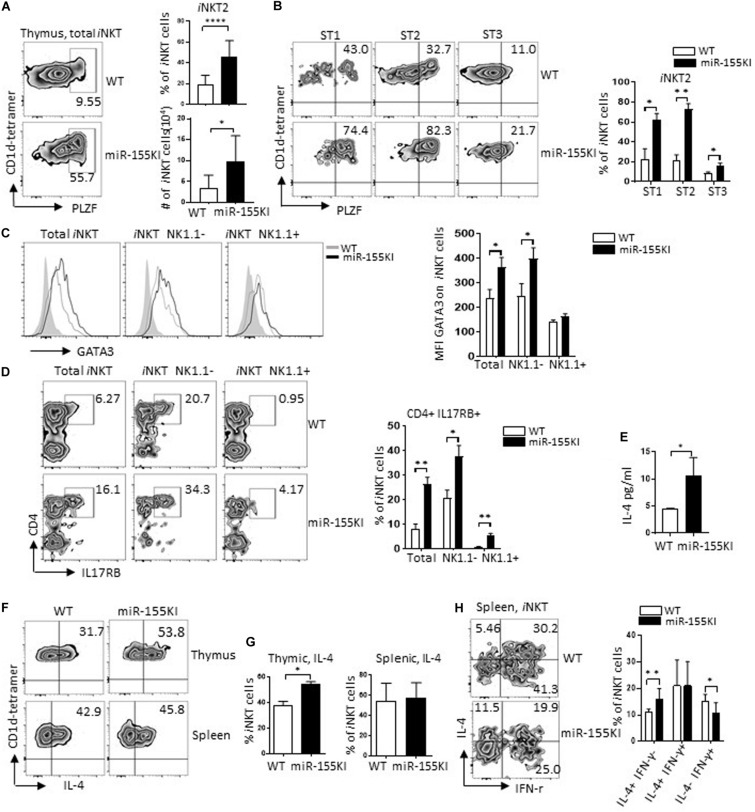
MiR-155 overexpression promotes *i*NKT2 cell differentiation. **(A)** Representative flow cytometry plots depicting *i*NKT2 (PLZF^hi^) in total thymic *i*NKT cells from miR-155KI and WT mice (left). Bar graphs showing frequency and absolute number of *i*NKT2 cells from miR-155KI and WT mice (right). **(B)** Representative flow cytometry plots depicting PLZF^hi^ *i*NKT cells (*i*NKT2) in different developmental stages of *i*NKT cell development from miR-155KI and WT mice (left). Bar graphs showing frequency of PLZF^hi^ *i*NKT cells (*i*NKT2) in stage 1 (ST1), stage 2 (ST2), and stage 3 (ST3) of *i*NKT cells from miR-155KI mice and WT controls (right). **(C)** Representative flow histogram depicting of GATA3 expression in total *i*NKT cells, NK1.1^–^, and NK1.1^+^ *i*NKT cells from miR-155KI (black line without shading) and WT (gray line without shading) mice (left). Bar graph showing mean fluorescence intensity (MFI) of GATA3 in indicated *i*NKT subsets from miR-155KI and WT mice. **(D)** Representative flow cytometry plots depicting CD4^+^ IL17RB^+^ *i*NKT cells, total *i*NKT cells, NK1.1^–^ and NK1.1^+^ *i*NKT cells from miR-155KI and WT mice (left). Bar graph showing frequency of CD4^+^ IL17RB^+^ *i*NKT cells in indicated *i*NKT subsets from miR-155KI and WT mice. **(E)** Bar graph showing IL-4 secretion in serum in miR-155KI and WT mice from one of two experiments and three mice from each group. **(F)** Representative flow cytometry plots depicting IL-4 production in thymic *i*NKT cells (top) and splenic *i*NKT cells (bottom) from miR-155KI and WT mice. **(G)** Bar graph shows frequency of IL-4 producing *i*NKT cells in thymus (left) and spleen (right) from miR-155KI and WT mice. **(H)** Representative flow cytometry plots depicting IL-4 and IFN-γ production in splenic *i*NKT cells from miR-155KI and WT mice. Bar graph shows frequencies of IL4^+^ IFN-γ^–^, IL-4^+^ IFN-γ^+^, and IL4-IFN-γ^+^ splenic *i*NKT cells from miR-155KI and WT mice. Data were from five independent experiments and analyzed by unpaired *t*-test. *P < 0.05, **P < 0.01, ****P < 0.0001.

*i*NKT2 cells (PLZF^hi^) are typically located in stages 1/2 *i*NKT cell pool. Compared with WT, thymic *i*NKT cells from miR-155KI mice exhibited a significant increased PLZF level (Figure 4A). However, the increased level of PLZF could merely reflect an increased frequency of *i*NKT cells stuck at stages 1/2 (Figures 2C,D). To address this possibility, we evaluated PLZF expression at different developmental stages of *i*NKT cells. We found that PLZF was increased in all individual developmental stages (Figure 4B), ruling out the possibility that the increased PLZF level could be secondary to the accumulation of cells in stages 1/2, indicating that miR-155 overexpression promotes PLZF expression during *i*NKT cell development. Consistently, other *i*NKT2 cell signatures including GATA3 (*Gata3*) and IL17RB (*Il17rb*) (Lee et al., 2013; Kwon and Lee, 2017) were also increased in miR-155KI *i*NKT cells, especially in the stage 1/2 (NK1.1^–^) *i*NKT cell pool (Figures 4C,D). Given that *i*NKT2 cells in steady status have the ability to produce copious amounts of IL-4 (Lee et al., 2013), we then evaluated serum IL-4 level *via* ELISA. As expected, serum IL-4 levels was increased in miR-155KI mice (Figure 4E). In addition, upon PMA/Ionocymin *in vitro* stimulation, IL-4 production from thymic *i*NKT cells was significantly increased in miR-155KI mice compared with WT controls. However, IL-4 production was similar in splenic *i*NKT cells (Figures 4F,G). Even though a majority of *i*NKT2 cells produce IL-4, mature *i*NKT1 cells have the ability to produce IL-4 and IFN-γ simultaneously, and the comparable IL-4 production could be due to the compensatory result from dramatically increased *i*NKT2 and reduced *i*NKT1 cells in the miR-155KI spleen. In support of this possibility, we found that splenic *i*NKT cells producing only IL-4 were increased, while IL-4 and IFN-γ double producing splenic *i*NKT cells were reduced in miR-155KI, even though the overall IL-4 producing splenic *i*NKT cells were comparable between miR-155KI and WT mice (Figure 4H). The *i*NKT cell maturation defect that was identified in PLZF transgenic mice closely resembles the defective *i*NKT1 but elevated *i*NKT2 differentiation phenotypes that we observed in our miR-155KI mice (Savage et al., 2008). Therefore, our data indicate that miR-155 overexpression elevates PLZF expression, promoting *i*NKT2 differentiation but blocking *i*NKT1 differentiation and maturation.

### MiR-155 Overexpression Interrupts *i*NKT17 Cell Differentiation

**FIGURE 5:**
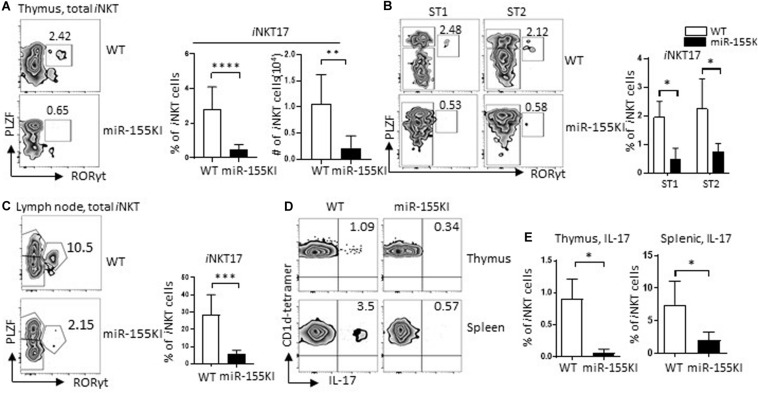
MiR-155 overexpression interrupts *i*NKT17 cell differentiation. **(A)** Representative flow cytometry plots depicting *i*NKT17 (PLZF^int^ RORγt^+^) in total *i*NKT cells from miR-155KI and WT mice (left). Bar graphs showing frequency and absolute number of *i*NKT17 cells from miR-155KI and WT mice (right). **(B)** Representative flow cytometry plots depicting *i*NKT17 cells in stages 1 (ST1) and 2 (ST2) of *i*NKT cells from miR-155KI and WT mice (left). Bar graph showing frequency of *i*NKT17 cells in ST1 and ST2 *i*NKT cells from miR-155KI and WT mice (right). **(C)** Representative flow cytometry plots depicting *i*NKT17 (PLZF^int^ RORγt^+^) in lymph node *i*NKT cells from miR-155KI and WT mice (left). Bar graph showing frequency of *i*NKT17 cells in lymph node from miR-155KI and WT mice (right). **(D)** Representative flow cytometry plots depicting IL-17 production in thymic *i*NKT cells (top) and splenic *i*NKT cells (bottom) from miR-155KI and WT mice. **(E)** Bar graphs showing frequency of IL-17 producing *i*NKT cells in thymus (left) and spleen (right) from miR-155KI and WT mice. Data are from at least three independent experiments. Data were analyzed by unpaired *t*-test. *P < 0.05, **P < 0.01, ***P < 0.001, ****P < 0.0001.

*i*NKT17 is a distinct subset of *i*NKT cells characterized by the expression of transcription factor RORγt (*Rorc*) (Constantinides and Bendelac, 2013). These cells differ from their classical *i*NKT1/2 counterparts by undergoing an alternative thymic pathway. The developmental stage at which *i*NKT17 cells and *i*NKT1/2 cells drift apart seems to be as early as CD4^+^CD8^+^ DP stage 0 (Michel et al., 2008). Here, we found that *i*NKT17 cells were dramatically reduced in miR-155KI mice, which can be observed at as early as stage 1 (Figures 5A,B). We then evaluated *i*NKT17 cells in lymph nodes due to the dramatic enrichment of *i*NKT17 cells in lymph node tissues. Consistent with the thymic *i*NKT17 phenotype, miR-155KI mice showed diminished *i*NKT17 cell frequencies in lymph nodes (Figure 5C). In support of the diminished thymus and peripheral *i*NKT17 phenotypes, IL-17 production induced by *in vitro* PMA/ionomycin stimulation was reduced significantly in both thymus and spleen *i*NKT cells from miR-155KI cells compared with WT (Figures 5D,E). These data indicate that overexpression of miR-155 interrupts *i*NKT17 lineage differentiation and IL-17 production.

### MiR-155 Overexpression Regulates Innate CD8 T Cell Development

**FIGURE 6:**
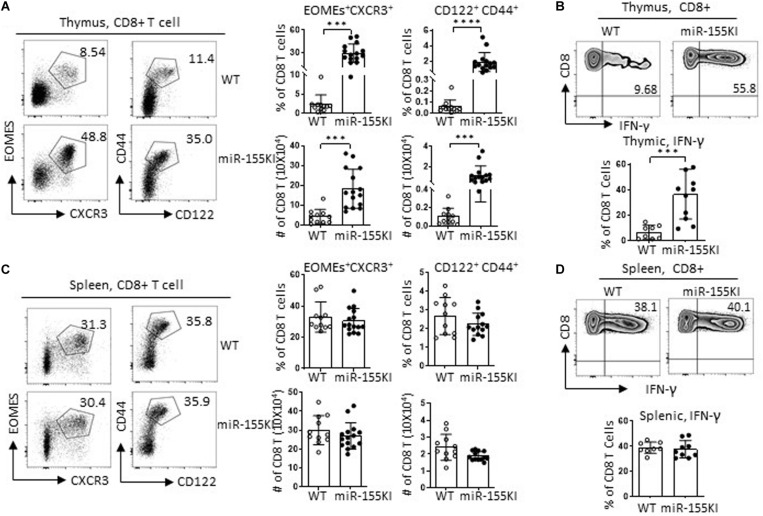
MiR-155 overexpression regulates innate CD8 T cell development. **(A,C)** Representative flow cytometry plots depicting innate CD8 T cells in thymic CD8 T cells and splenic CD8 T cells; CD8 T cells gating strategy are shown in Supplementary Figure 4. Innate CD8 T cells were identified as CXCR3^+^Eomes^+^ and CD44^hi^CD122^+^ left in thymus **(A)** and spleen **(C)**. Bar graphs showing frequency and absolute number of innate CD8 T cells in thymus **(A)** and spleen **(C)** from miR-155KI and WT mice (right). **(B,D)** Representative flow cytometry plots depicting IFN-γ production in thymic **(B)** and splenic **(D)** CD8^+^ T cells from miR-155KI and WT mice. Bar graphs showing frequency of IFN-γ^+^ CD8 T cells in thymus **(B)** and spleen **(D)** from miR-155KI and WT mice. Data are from at least four independent experiments. Each individual mouse is represented by a dot, and the data were analyzed by non-paired *t*-test. ***P < 0.001, ****P < 0.0001.

The CD4Cre-mediated gene mutation occurred not only in *i*NKT cells and precursors but also in conventional T cells. To evaluate the role of miR-155 overexpression in conventional T cell development, we checked on the gross conventional T cell phenotypes with flow cytometry. Interestingly, miR-155KI mice showed increased frequencies and numbers of both CD8 single positive (CD8 SP) and CD4 SP T cells but decreased frequencies and numbers of DP T cells (Supplementary Figure 4A). Further analysis revealed increased regulatory T cell (Treg) frequencies in thymus CD4 SP T cells from miR-155KI mice compared with WT (Supplementary Figure 4B), which is consistent with a previous report (Kohlhaas et al., 2009). More interestingly, thymic CD8 SP T cells from miR-155KI mice contained significantly increased innate CD8 T cells, characterized by a relative high expression of CD44, CD122, CXCR3, and Eomes (Figure 6A and Supplementary Figure 4A), and dramatically higher IFN-γ production upon PMA/ionocymin stimulation (Figure 6B). However, conventional CD8 and innate CD8 T cell phenotypes were only observed in thymus and not in spleen T cells from miR-155 KI mice (Figures 6C,D and Supplementary Figure 4C). Considering that innate CD8 T cells are barely detectable in WT C57BL/6 thymus, their presence in miR-155KI could be attributed to the expansion of small numbers of IL-4-producing *i*NKT2 cells observed in miR-155KI mice.

### Cell-Intrinsic Versus Extrinsic Effects of Defective *i*NKT Cells and Innate CD8 T Cells With MiR-155 Overexpression

The development of *i*NKT cells depends on positive selection from signals through glycolipids presented on DP thymocytes by the CD1d, as well as through homotypic interactions with the SLAM family receptors Slamf1 and Slamf6 (Griewank et al., 2007). In addition to the intrinsic factors of the *i*NKT precursors, the DP T cell-constituted thymic environmental changes induced by miR-155 overexpression may also have an impact on *i*NKT cell development and differentiation. Here, we found that CD1d, Slamf1, and Slamf6 expression were comparable in thymic DP T cells between miR-155KI and WT mice (Supplementary Figure 5A).

**FIGURE 7:**
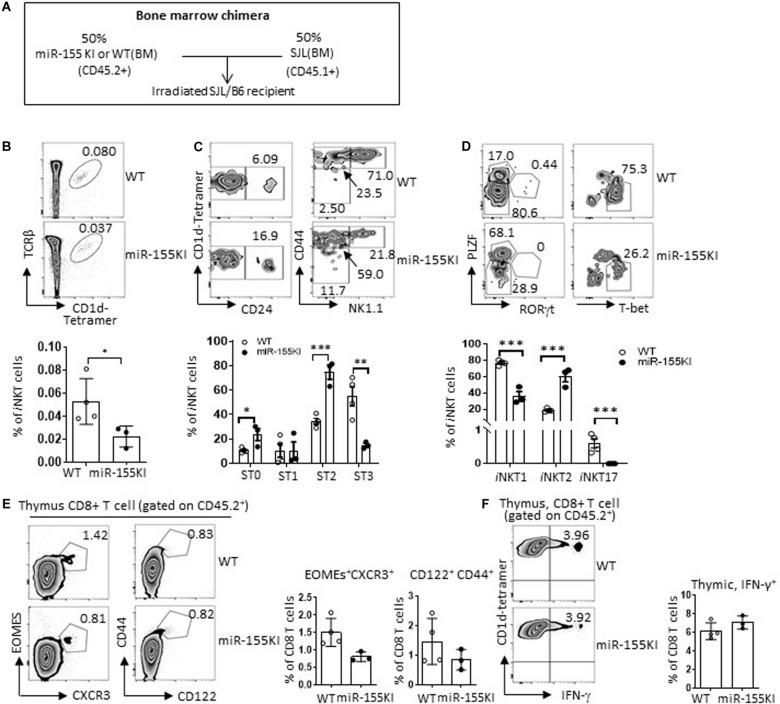
Cell-intrinsic versus extrinsic effects of defective *i*NKT cell with miR-155 overexpression. **(A)** Schematic representation of the mixed bone marrow chimera. **(B–D)** Donor bone marrows (BM) harvested from age- and gender-matched SJL (CD45.1^+^) mice and miR-155KI (CD45.2^+^) or WT (CD45.2^+^) mice with CD3 deletion were cotransferred at 1:1 ratio to 8-week-old B6.SJL recipient mice that were lethally irradiated. **(B)** Representative flow cytometry plots depicting *i*NKT cells in thymus derived from CD45.2^+^ WT and CD45.2^+^ miR-155KI BM (top), CD45.1 and CD45.2 gating strategy are provided in Supplementary Figure 5B. Bar graph showing frequency of *i*NKT cells derived from CD45.2^+^ WT and CD45.2^+^ miR-155KI BM (bottom). **(C)** Flow cytometry plots depicting the stage 0 (ST0, CD24^+^), stage 1 (ST1, CD24^–^ CD44^lo^NK1.1^–^), stage 2 (ST3, CD24^–^ CD44^hi^NK1.1^–^), and stage 3 (ST3, CD24^–^ CD44^hi^NK1.1^+^) gated on thymic *i*NKT cells. Bar graph showing frequencies of different stages of *i*NKT cells derived from CD45.2^+^ WT and CD45.2^+^ miR-155KI BM (bottom). **(D)** Flow cytometry plots depicting *i*NKT1 (PLZF^lo^T-bet^hi^), *i*NKT2 (PLZF^hi^), and *i*NKT17 (PLZF^int^RORγt^+^) cells (top). Bar graph showing frequencies of different subsets of *i*NKT cells derived from CD45.2^+^ WT and CD45.2^+^ miR-155KI BM (bottom). **(E)** Representative flow cytometry thymic innate CD8 T cells (CXCR3^+^Exomes^+^ and CD44^hi^CD122^+^) derived from CD45.2^+^ WT and CD45.2^+^ miR-155KI BM (left). Bar graph shows frequency of innate CD8 T cells derived from CD45.2^+^ WT and CD45.2^+^ miR-155KI BM (right). **(F)** Representative flow cytometry depicting IFN-γ production in thymic CD8 T cells derived from CD45.2^+^ WT and CD45.2^+^ miR-155KI BM (left). Bar graph showing frequency of IFN-γ^+^ thymic CD8 T cells derived from CD45.2^+^ WT and CD45.2^+^ miR-155KI BM (right). Data are from three independent experiments. Each individual mouse is represented by a dot, and the data were analyzed by unpaired *t*-test. *P < 0.05, **P < 0.01, ***P < 0.001.

To further rule out the possibility of a thymic environmental defect in miR-155KI mice, we performed a mixed bone marrow (BM) chimera experiment. A 1:1 mixture of SJL (CD45.1^+^) BM with miR-155KI or WT littermate BM (CD45.2^+^) was transferred to lethally irradiated SJL/B6-recipient mice (CD45.1^+^CD45.2^+^) (Figure 7A). Eight weeks post-BM transfer, *i*NKT and T cell constitution and phenotypes were evaluated and compared. We found that *i*NKT cells that had originated from the miR-155 KI BM perfectly mimicked the defective *i*NKT cell phenotypes identified in primary miR-155 KI mice, as seen by *i*NKT cell frequency, maturation status, and functional lineage differentiation (Figures 7B–D). These results showed conclusively that the observed defects in *i*NKT cell development and differentiation were entirely cell intrinsic.

On the other hand, thymic CD8 SP T cells that had originated from miR-155KI BM showed an indistinguishable phenotype compared with WT BM based on the CD8 SP T frequencies, innate CD8 T cell marker expression, and IFN-γ production upon PMA/Ionomycin *in vitro* stimulation (Figures 7E,F). Therefore, the role of miR-155 in thymic innate CD8 T cell development is entirely cell extrinsic. This cell extrinsic feature of thymic innate CD8 T cell development is in perfect agreement with the observed enhanced thymic *i*NKT2 differentiation and IL-4 production, which is supported by multiple previous studies that showed that defective *i*NKT cell end-stage maturation is accompanied by an enhanced thymic innate CD8 T cell development (Lee et al., 2011).

### MiR-155 Regulates *i*NKT Cell and Innate CD8 T Cell Development by Targeting Multiple Genes

**FIGURE 8:**
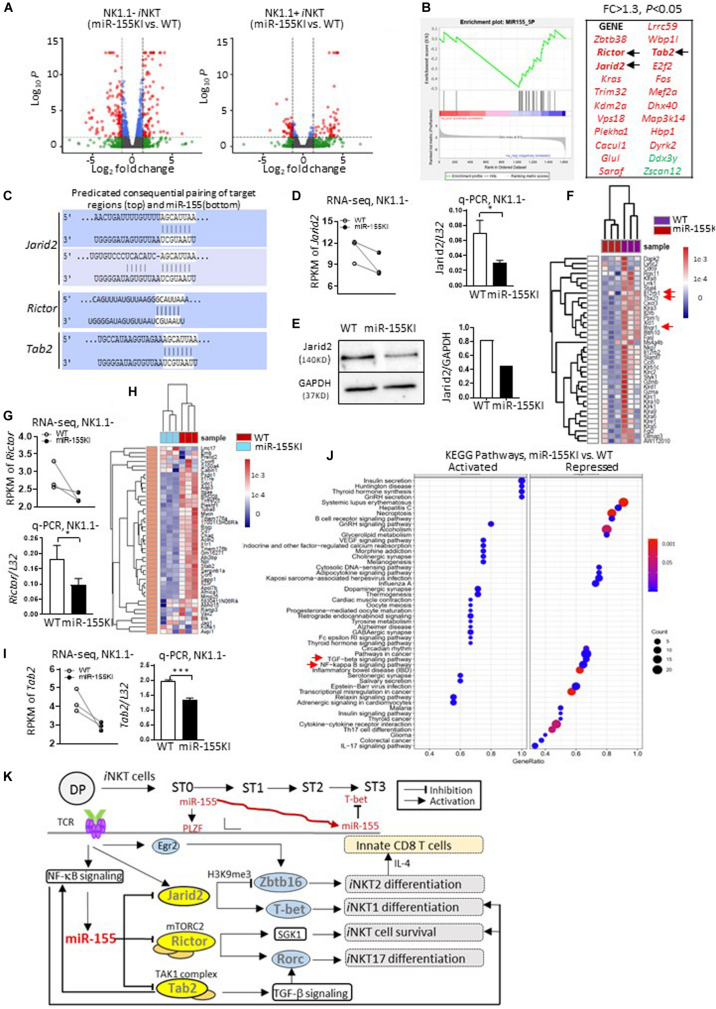
MiR-155 regulates *i*NKT cell and innate CD8 T cell development by targeting multiple genes. **(A)** Volcano plot shows significant differentially expressed genes from NK1.1^–^ (miR-155 KI vs. WT, left) and NK1.1^+^ (miR-155 KI vs. WT, right) RNA pools. **(B)** GSEA plot demonstrates enrichment of miR-155 target genes in miR-155KI cells. The *x*-axis represents the rank ordering (miR-155KI vs. WT) of all genes. A running GSEA enrichment score for miR-155 target genes is plotted along the rank order. MiR-155 target genes are individually identified with a black tick marker at their rank positions (also listed on the right in red). **(C)** The schematic representation of *Jarid2* (top), *Rictor*, and *Tab2* (bottom) binding sites with miR-155. **(D)** RPMK of *Jarid2* differences in WT and miR-155KI *i*NKT cells from RNA-seq. Each dot represents one sample (left). *Jarid2* relative expression in WT and miR-155KI *i*NKT cells from q-PCR (right). **(E)** Western blot analysis of Jarid2 protein in total thymocytes from miR-155KI and WT mice (left). Cropped images of Jarid2 and GAPDH have been used and the full scans of the entire original blots are available in the Supplementary Figure 6. Bar graph shows relative Jarid2 expression in miR-155 KI and WT mice (right). The experiment was reproduced two times with similar results. **(F)** Heat map comparing gene signatures for *i*NKT1 in *i*NKT cells from miR-155KI and WT mice: among these, *Il12rb1*, *Tbx21*, and *Ifngr1* are highlighted with red arrows. **(G)** RPMK of *Rictor* differences in WT and miR-155KI *i*NKT cells from RNA-seq. Each dot represents one sample (top). *Rictor* relative expression in WT and miR-155KI *i*NKT cells from q-PCR (bottom). **(H)** Heat map comparing gene signatures for *i*NKT17 in *i*NKT cells from miR-155KI and WT mice. **(I)** RPMK of *Tab2* differences in WT and miR-155KI *i*NKT cells from RNA-seq. Each dot represents one sample (left). *Tab2* relative expression in WT and miR-155KI *i*NKT cells from q-PCR (right) and data were analyzed by unpaired t-test. *P < 0.05, ***P < 0.001. **(J)** KEGG pathway analysis in NK1.1^–^ RNA pools (miR-155KI vs. WT). Left panel shows activated signaling pathways in miR-155KI *i*NKT cells; right panel showing repressed signaling pathways in miR-155KI *i*NKT cells. The TGF-β signaling pathway and NF-κB signaling pathway are highlighted with red arrows. **(K)** A schematic, speculative model of miR-155 regulation of *i*NKT cell and innate CD8 T cell development through targeting of multiple signaling molecules and pathways. miR-155, which is probably induced by TCR activation *via* NF-κB signaling, is gradually downregulated during *i*NKT cell maturation. At early stages, high levels of miR-155 further promote PLZF expression (*via* repressing *Jarid2*) on the basis of high levels of PLZF, which was initially induced by Egr2. Additionally, as *i*NKT cells develop, reduced miR-155 could release the repression of T-bet (*via* relieving *Jarid2*). On the other hand, miR-155 simultaneously inhibits Tab2 and NF-κB signaling pathways, therefore forming a negative feedback loop controlling *i*NKT1/2 differentiation. Meanwhile, reduced miR-155 could relieve the suppression of Rictor and TGF-β signaling pathways, which cooperate additively for the differentiation of the *i*NKT17 lineage and regulation of overall *i*NKT homeostasis.

To identify the target molecules of miR-155 during *i*NKT thymic development and differentiation, we then performed an unbiased RNA-Seq analysis of sorted stage1/2 NK1.1^–^ *i*NKT cells and stage 3 NK1.1^+^ *i*NKT cells from miR-155KI and WT littermate mice. Differential gene expression analysis revealed 248 down- and 234 upregulated genes (among 11,636 variables) in the NK1.1^–^ *i*NKT cell pool, and 26 down- and 52 upregulated genes (among 10,263 variables) in the NK1.1^+^ *i*NKT cell pool (Figure 8A). A vast number of genes in the NK1.1^–^ *i*NKT cell pool were dysregulated.

To confirm a global dysregulation of miR-155 targets in miR-155KI mice, a FPKM-rank-ordered (miR-155KI vs. WT) list of genes was prepared and used as input for a preranked GSEA analysis (Subramanian et al., 2005) against an MsigDB miRNA target gene set (C3 miRNA v3.0; MSigDB). The significant association (*p* < 0.05; Kolmogorov–Smirnov test) was identified for genes within the miR-155 target gene set and confirmed a transcriptome-wide reduction of gene expression in miR-155KI NK1.1^–^ and NK1.1^+^ *i*NKT cell pools. With this strategy, we identified 21 predicted miR-155 targets that were significantly repressed in miR-155KI NK1.1^–^ *i*NKT cells (Figure 8B and Supplementary Table 1). However, no predicted miR-155 targets were identified in the NK1.1^+^ *i*NKT cell pool (data not shown). It is important to point out that neither *Itk* nor *Ets1* were in the downregulated gene list for both NK1.1^–^ and NK1.1^+^ *i*NKT cells in miR-155KI mice, even though both were previously reported as potential targets of miR-155 during *i*NKT cell development (Burocchi et al., 2015). Therefore, in subsequent experiments, we focused on the NK1.1^–^ *i*NKT cell pool for mechanistic analysis. Among these predicted target genes, we were particularly intrigued by the genes *Jarid2*, *Rictor*, and *Tab2*, all of which were shown to be significantly decreased in miR-155KI *i*NKT cells based on RNA-Seq data (Figure 8B). In support of the roles of *Jarid2*, *Rictor*, and *Tab2* as targets of miR-155, there are two predicted miR-155 binding sites in the 3′ UTR of *Jarid2*; one predicated miR-155 sites in the 3′ UTR of *Rictor*; and one predicated miR-155 sites in the 3′ UTR of *Tab2* (Dianalab algorithm, Figure 8C).

*Jarid2* is a member of the JmjC domain-containing protein family, a novel component of polycomb repressive complex 2 (PRC2). A previous study confirmed that *Jarid2* is a negative regulator of *Zbtb16* (encode PLZF) mediated by H3K9me3 demethylation (Pereira et al., 2014). Indeed, *i*NKT cells from miR-155KI mice showed down regulated expression of *Jarid2*, which was verified *via* q-PCR (Figure 8D), but increased expression of PLZF (Figures 4A,B). In support of decreased *Jarid2* expression in *i*NKT cells, thymocytes from miR-155KI mice showed a pronounced reduction in Jarid2 protein levels (Figure 8E and Supplementary Figures 6A,B). More importantly, the *i*NKT phenotype of T cell-specific *Jarid2* deletion closely resembled what was observed in miR-155KI mice, as judged by the disturbed *i*NKT1/2 cells differentiation and increased thymic innate CD8 T cell development (Pereira et al., 2014). Consistently, RNA-seq showed that a cluster of *i*NKT1 signature genes were downregulated in miR-155KI *i*NKT cells (Figure 8F). Among these, we found that *i*NKT cells with miR-155 KI showed interrupted *Ifngr1* and *Il12rb1* expression (Figure 8F). Given that IFN-γ and IL-12 signaling promote T-bet expression during Th1 differentiation (Zhu et al., 2012), the impaired IFN-γ and IL-12 signaling pathway may be the direct molecular mechanism for the interrupted T-bet expression and *i*NKT1 differentiation.

These data suggested that miR-155 inhibits *Jarid2*, which negatively regulates PLZF expression to control *i*NKT development, *i*NKT1/2 lineage differentiation, and secondary innate CD8 T cell development. Even though the data overwhelmingly supported the dominant target role of *Jarid2* in the defective *i*NKT cell phenotypes in miR-155KI mice, the downregulation of *Jarid2* does not explain all the *i*NKT phenotypes observed in miR-155KI mice, such as defective *i*NKT cell survival and diminished *i*NKT17 lineage differentiation. As one miRNA could target multiple genes in one cell setting, *Rictor* attracted our attention as another potential target of miR-155 in *i*NKT cells (Figures 8B,C). *Rictor* is an obligatory component of mechanistic target of rapamycin (mTOR) complex 2 mTORC2 (Laplante and Sabatini, 2009). Here, we found that *Rictor* expression was dramatically reduced in miR-155KI *i*NKT cells, and this result was verified *via* q-PCR (Figure 8G). The deletion of *Rictor* selectively abolished the *i*NKT17 lineage, as indicated by a marked reduction of RORγt and IL-17 expression (Wei et al., 2014; Sklarz et al., 2017). Alternatively, *i*NKT cells with *Rictor* deletion have been shown to have defective survival during development (Sklarz et al., 2017). Interestingly, these defective *i*NKT phenotypes are highly reminiscent of what was observed in miR-155KI mice, particularly with regard to diminished *i*NKT17 lineage differentiation. Of interest, the global *i*NKT17 signature genes were significantly downregulated in miR-155KI *i*NKT cells (Figure 8H). Strikingly, the expression of *Sgk1*, a cell survival kinase and the downstream molecule of Rictor/mTORC2 (Lang and Cohen, 2001; Loffing et al., 2006), was found to be significantly reduced in miR-155KI *i*NKT cells, as determined by RNA-Seq (data not shown). These data strongly support the idea that miR-155 inhibits *i*NKT17 lineage differentiation and controls *i*NKT survival through targeting *Rictor*. Thus, downregulation of miR-155 to derepress *Rictor* during *i*NKT thymic development may be necessary for overall *i*NKT development and *i*NKT17 lineage differentiation.

Furthermore, we found that *Tab2* (TGF-β activated kinase 1-binding protein 2), one of the predicted targets of miR-155, was also repressed in miR-155 KI *i*NKT cells (Figures 8B,C,I). *Tab2* is required for transforming growth factor-β-activated kinase 1 (TAK1)-dependent signaling pathways, including the transforming growth factor-β (TGF-β) signaling pathway and the NF-κB signaling pathway (Takaesu et al., 2000; Kanayama et al., 2004; Hu et al., 2014; Tan et al., 2015), both of which are critical for *i*NKT cell development at multiple levels (Sivakumar et al., 2003; Vallabhapurapu et al., 2008; Havenar-Daughton et al., 2012). In support of this notion, our KEGG pathway analysis revealed that both the NF-κB and the transforming growth factor-β (TGF-β) signaling pathways were partially inhibited in miR-155KI *i*NKT cells (Figure 8J and Supplementary Figures 7A,B). Taken together, it is fair to conclude that miR-155 regulates *i*NKT1/2 cell differentiation and secondary innate CD8 T cell development by targeting *Jarid2* and controls *i*NKT17 differentiation and *i*NKT homeostasis by targeting *Rictor.* Additionally, miR-155 modulates the NF-κB signaling pathway and TGF-β signaling pathway during *i*NKT cell maturation and cell function (Figure 8K).

## Discussion

MiR-155 was one of the first discovered mammalian miRNAs and is expressed in a variety of immune cells. Extensive studies have revealed the complex and versatile functions of miR-155 in different immune cells. In B cells, miR-155 overexpression leads to the transformation and development of lymphoma (Costinean et al., 2006), which is correlated with the role of miR-155 in human hematopoietic cancers (Eis et al., 2005; Kluiver et al., 2007). In T cells, miR-155 has been reported to regulate cell lineage decisions, where it suppresses Th2 differentiation (Rodriguez et al., 2007), promotes autoimmune inflammation by enhancing inflammatory T cell development (O’Connell et al., 2010), enhances the effector CD8 T cell immunity, and restrains central memory CD8 differentiation in response to viruses and during cancer (Almanza et al., 2010; Tsai et al., 2013). In the current study, we showed the essential role of dynamic miR-155 expression in the development, differentiation, and homeostasis of *i*NKT cells. Overexpression of miR-155 perturbed *i*NKT1 but promoted *i*NKT2 differentiation by targeting *Jarid2*, the component of a negative epigenetic regulator of PLZF, while miR-155 restrained *i*NKT17 differentiation by targeting *Rictor*, an obligatory component of mTORC2. Our discovery adds further complexities of miR-155-mediated immune regulation and emphasizes the cell type, developmental, and differentiation stage-specific regulation characteristics and molecular mechanisms of miR-155-mediated immune regulation.

PLZF is a signature transcription factor of the *i*NKT cell lineage. PLZF directs the *i*NKT cell effector program, lineage differentiation, and function and affects cell migratory properties (Kovalovsky et al., 2008; Savage et al., 2008). Previous studies have revealed the molecular network regulating PLZF expression. Egr2, one of the earliest transcription factors induced by TCR signaling, directly binds to and activates the PLZF promoter, resulting in PLZF expression that provides a link between TCR signaling and cell fate determination in *i*NKT cells (Seiler et al., 2012). During thymic *i*NKT development, the dynamic upregulation of let-7 miRNA is involved in the gradual downregulation of PLZF and *i*NKT1 differentiation and maturation (Pobezinsky et al., 2015). Our current study indicates that miR-155 regulates *i*NKT cell lineage differentiation through targeting *Jarid2*, which epigenetically restricts the expression of PLZF, further expanding the complexity of the molecular networks in controlling PLZF and *i*NKT cell differentiation and maturation. Previous studies have indicated that either Egr2 or Jarid2 and miR-155 are upregulated by TCR activation (Seiler et al., 2012; Pereira et al., 2014), and that Jarid2 and Egr2 may counter-regulate PLZF expression in *i*NKT cells. Nevertheless, the magnitude of the exponential surge of Egr2 expression far outweighs the slight upregulation of *Jarid2* after TCR activation, which indicates the dominant regulatory role of Egr2 in the initial PLZF transcriptional boost and in the early stages of *i*NKT cell development and effector programs (Pereira et al., 2014). However, epigenetic regulation, including *Jarid2* elevation resulting from miR-155 downregulation and IL-15-promoted Let-7 expression are critically involved in the gradual downregulation of PLZF, which is essential for *i*NKT1 differentiation and maturation (Pobezinsky et al., 2015). These may also explain why knockdown of miR-155 does not significantly interrupt PLZF expression or development and differentiation of *i*NKT cells, in which miR-155 deletion-mediated *Jarid2* elevation may not be enough to perturb the early PLZF boost dominated by the Egr2 surge.

MiR-155 transcription was reported to be positively regulated through NF-κB signaling pathway. Therefore, the upregulation of miR-155 after TCR activation during the early *i*NKT developmental stage may be mediated through the NF-κB signaling pathway. Nevertheless, *i*NKT cells with miR-155 overexpression showed downregulated NF-κB signaling pathway, possibly through directly targeting *Tab2*, the upstream activation kinase complex component of NF-κB (Kanayama et al., 2004; Gatto et al., 2008). Nevertheless, a positive regulatory role of miR-155 on NF-κB signaling pathway was reported in a B cell lymphoma cell line, which may indicate the cell-context-specific regulatory role of miR-155 (Jablonska et al., 2015). Furthermore, mTORC2 positively regulates NF-κB signaling pathway through AKT (Sarbassov et al., 2005; Cheng et al., 2011). Hence, miR-155 can suppress the NF-κB signaling pathway indirectly through targeting *Rictor*. The NF-κB signaling pathway is known to be critical for *i*NKT cell differentiation. NF-κB-deficient mice, including NF-κB1, c-Rel, and RelA KO mice, have shown perturbed *i*NKT maturation: especially in RelA KO mice (Stankovic et al., 2011), which closely resembles the defective *i*NKT1 phenotypes in miR-155KI mice, particularly with regard to interrupted stage 3. Thus, the defective NF-κB signaling pathway in *i*NKT cells with miR-155 overexpression may cooperate additively with the perturbed *Jarid2* and derepression of PLZF in the overall defective *i*NKT1 phenotype of miR-155KI mice. On the other hand, the cell-intrinsic NF-κB activity was shown to be essential for the survival of *i*NKT cells (Gatto et al., 2008), which could be an additional reason for the interrupted survival of *i*NKT cells with miR-155 overexpression. Furthermore, the interaction between miR-155 and the NF-κB signaling pathway forms a negative feedback loop, which could be the reason for the transient or self-limiting manner of miR-155 expression induced by TCR activation, such as the gradual downregulation of miR-155 during *i*NKT thymic development and differentiation identified in the current study.

mTOR signaling integrates immune signals and metabolic cues in orchestrating T cell responses (Chi, 2012; Powell et al., 2012). mTOR signaling consists of two complexes, mTORC1 and mTORC2, which are defined by the respective signature components Raptor and Rictor. Previous studies from two groups have indicated a critical role of mTORC2 in the fate determination of *i*NKT17 cells and in *i*NKT homeostasis (Wei et al., 2014; Sklarz et al., 2017). Our results indicated that miR-155 inhibits *i*NKT17 lineage differentiation and controls *i*NKT survival through targeting *Rictor*, the required component of mTORC2. On the other hand, *i*NKT cells with miR-155 overexpression showed a downregulated TGF-β signaling pathway, while TGF-β signaling, particularly its SMAD4-dependent pathway, is required for both the survival of RORγt^+^ *i*NKT cells during their development and IL-17 production (Havenar-Daughton et al., 2012). Therefore, the perturbed mTORC2 activity and downregulated TGF-β signaling pathway may work additively for the defective *i*NKT17 differentiation and *i*NKT homeostasis in *i*NKT cells with miR-155 overexpression. Nonetheless, studies by Escobar et al. (2014) have shown that miR-155 positively regulates cytokine gene expression in Th17 cells through posttranscriptional regulation of *Jarid2*. These studies, combined with our data, emphasize the spatial, temporal, and delicately balanced characteristics of miR-155-mediated regulation. Our data also bring miRNAs and chromatin modification together by showing how miR-155 targets the chromatin protein Jarid2, which in turn regulates the key transcription factor for *i*NKT development and effector program, PLZF.

We do realize there are some limitations in our current study, including the dissection of role of miR-155 in *i*NKT and Treg cell interaction. The number of Tregs cells in thymus was found increased in miR-155KI mice (Supplementary Figure 4B). A previous report indicated that there is a potential across-talk between Tregs and *i*NKT cells and that Tregs in thymus may suppress the proliferation, cytokine production, and cytotoxic activity of *i*NKT cells in local environment (La Cava et al., 2006). Our current study is unable to rule out the potential role of Treg cells in the defective *i*NKT cell development and function, which need to be further investigated.

Overall, the dynamic expression of miR-155 and its regulation in thymic *i*NKT cells is essential for the development, functional lineage differentiation, and homeostasis of *i*NKT cells. Even though the early upregulation of miR-155 after *i*NKT positive selection is not a dominant factor for the initial PLZF transcription boost, the potential miR-155-NF-κB-negative feedback loop controlling miR-155 downregulation is essential for downstream *i*NKT differentiation and homeostasis. Downregulated miR-155 relief the suppression to *Jarid2* and the NF-κB signaling pathway, which further controls PLZF expression and balances *i*NKT1/*i*NKT2 differentiation cooperatively. On the other hand, downregulated miR-155 relieves the suppression of *Rictor* and the TGF-β signaling pathway, additively cooperating for the differentiation of the *i*NKT17 lineage and promotion of overall *i*NKT homeostasis. Consequently, the dynamic regulation of miR-155 expression during thymic *i*NKT cell development is essential, and miR-155 functions as a moderator connecting and coordinating multiple signaling pathways and transcriptional programs for a consolidated regulation of *i*NKT development, functional lineage differentiation, and secondary innate CD8 T cell development (Figure 8K).

## Data Availability Statement

The raw data supporting the conclusions of this article will be made available by the authors, without undue reservation, to any qualified researcher.

## Ethics Statement

The animal study was reviewed and approved by Experiments using animal were approved by the Institutional Animal Care and Use Committee of Henry Ford Health System.

## Author Contributions

LZ and Q-SM conceived and designed the study. JW, KL, XZ, GL, TL, and XW performed the experiments. JW, KL, and LZ analyzed the data. SB performed mouse irradiation for BM transfer. LZ, JW, and Q-SM wrote the manuscript, which was commented on by all authors.

## Conflict of Interest

The authors declare that the research was conducted in the absence of any commercial or financial relationships that could be construed as a potential conflict of interest.

## Footnotes

1 http://imagej.nih.gov/ij

2 www.r-project.org
