# Supplementary material for: MicroRNA-155 Controls iNKT Cell Development and Lineage Differentiation by Coordinating Multiple Regulating Pathways
Source: Front Cell Dev Biol. 2021 Jan 12;8:619220. doi: 10.3389/fcell.2020.619220 (PMC7874147; doi:10.3389/fcell.2020.619220)
Supplement: Supplementary file 1 [file Data_Sheet_1.PDF]

Supplemental Fig. 1

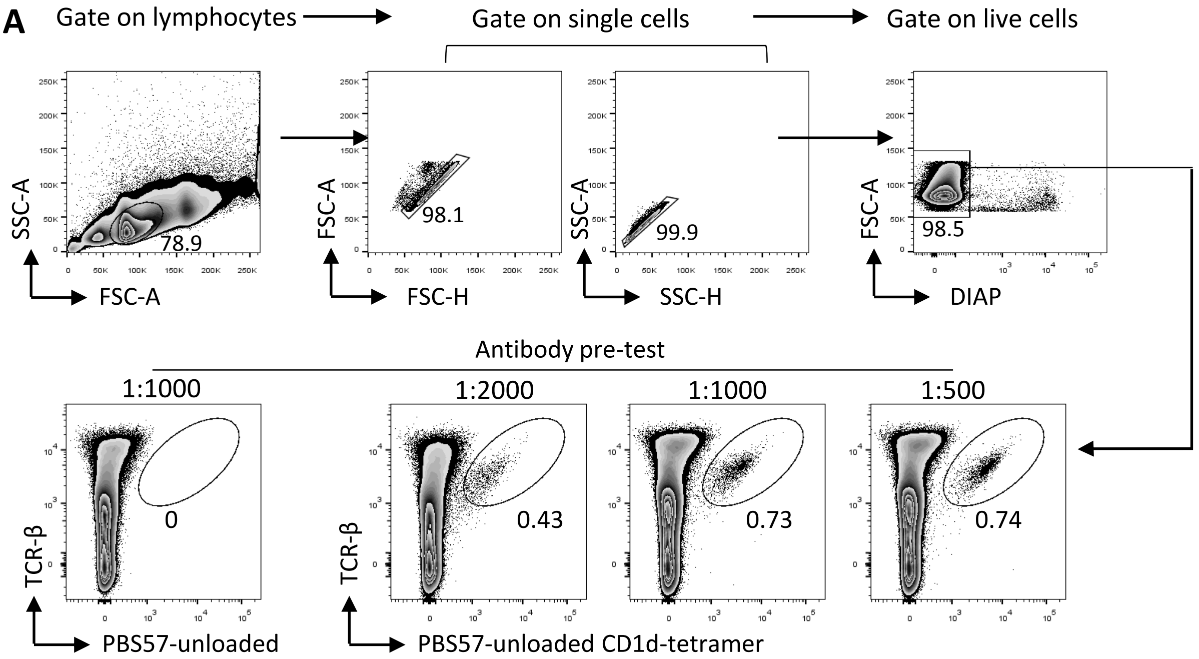

B

	Unstained Control	FMO Control			Fully Stained
FITC	----	TCR-β	TCR-β	TCR-β	TCR-β
PE	----	----	CD1d-Tetramer	CD1d-Tetramer	CD1d-Tetramer
PercP	----	CD8	----	CD8	CD8
APC	----	CD4	CD4	----	CD4

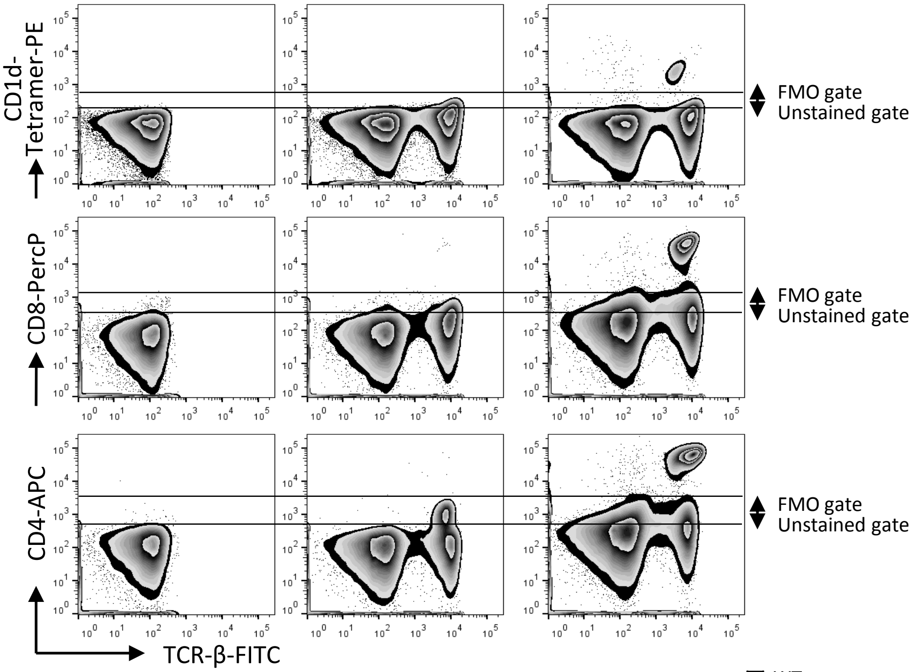

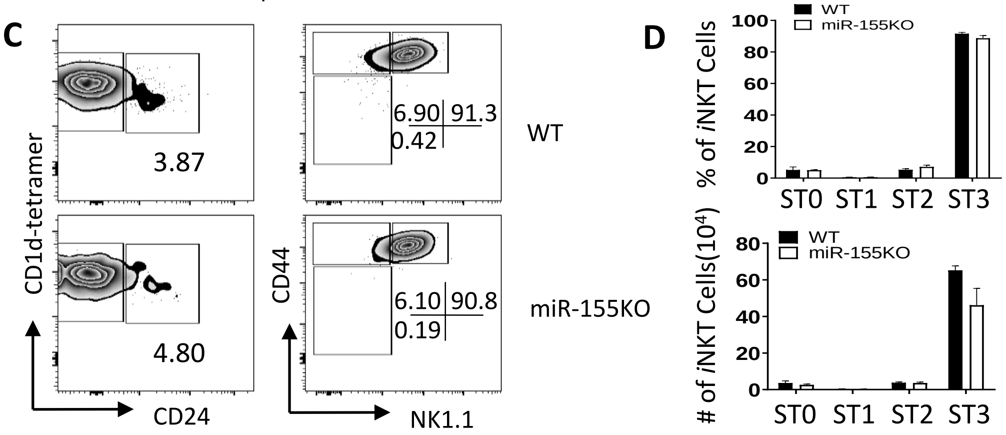

Supplemental Fig. 1 miR-155 deletion does not affect *i*NKT cell development

(**A**) Representative flow cytometry plots depicting lymphocytes gating strategy and CD1d-tetramer antibody pre-test results. (**B**) Fluorescence Minus One(FMO) control for iNKT cells, CD8 T cells, and CD4 T cells. (**C**)Representative flow cytometry plots depicting expression of CD24, CD44 and NK1.1 on *i*NKT cells. Bar graph showing frequency and absolute numbers of stage 0(ST0, $CD24^{+}$), stage 1 (ST1, $CD24^{-}CD44^{lo}NK1.1^{-}$), stage 2 (ST2, $CD44^{hi}$ $NK1.1^{-}$) and stage 3 (ST3, $CD44^{hi}$ $NK1.1^{+}$) in miR-155KO and WT mice. (**D**) Bar graphs showing frequencies (top) and absolute numbers (bottom) of *i*NKT cells in different developmental stages of miR-155KO and WT controls. Data are from two independent experiments; data were analyzed by unpaired *t*-test.

Supplemental Fig. 2

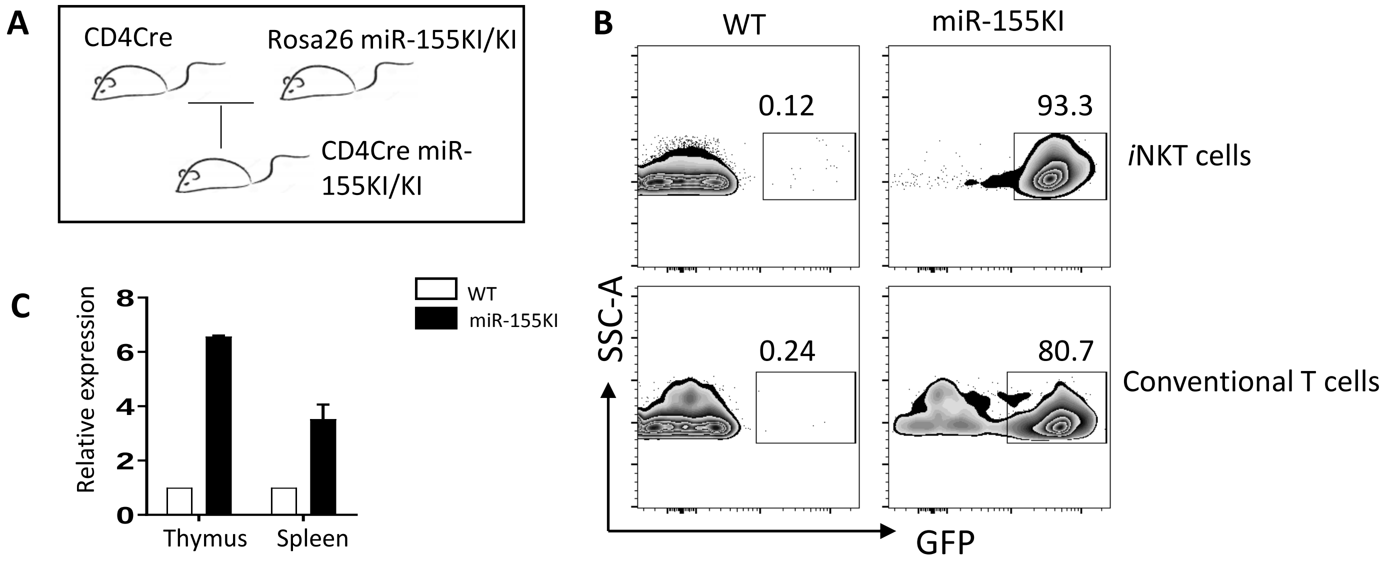

Supplemental Fig. 2 miR-155 overexpression mouse model

(**A**) CD4Cre miR-155 KI mouse model. (**B**) Representative flow cytometric plots depicting expression of GFP on thymic conventional T cells and *i*NKT cells from WT and miR155-KI (left); (**C**) Thymic T cells and splenic T cell were harvested from WT controls and miR-155KI mice. Total RNA was purified and miR-155 and snoRNU202 expression were measured by RT-qPCR.

Supplemental Fig. 3

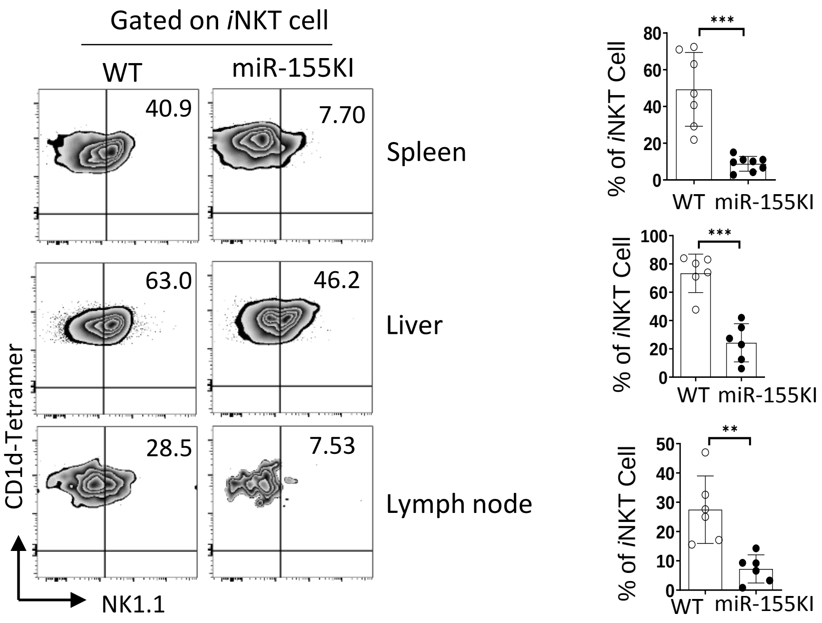

Supplemental Fig. 3   miR-155 overexpression interrupt peripheral *i*NKT cell maturation

Representative flow cytometric plots depicting expression of NK1.1 on *i*NKT cells from spleen, liver and lymph nodes (left); Bar graph showing frequency of $NK1.1^{+}$ *i*NKT cells in indicated organs from miR-155KI and WT mice (right).   Data are from three independent experiments. Each mouse is represented as one dot. Data were analyzed by unpaired *t*-test. ** $P < 0.01$, ***$P < 0.001$

Supplemental Fig. 4

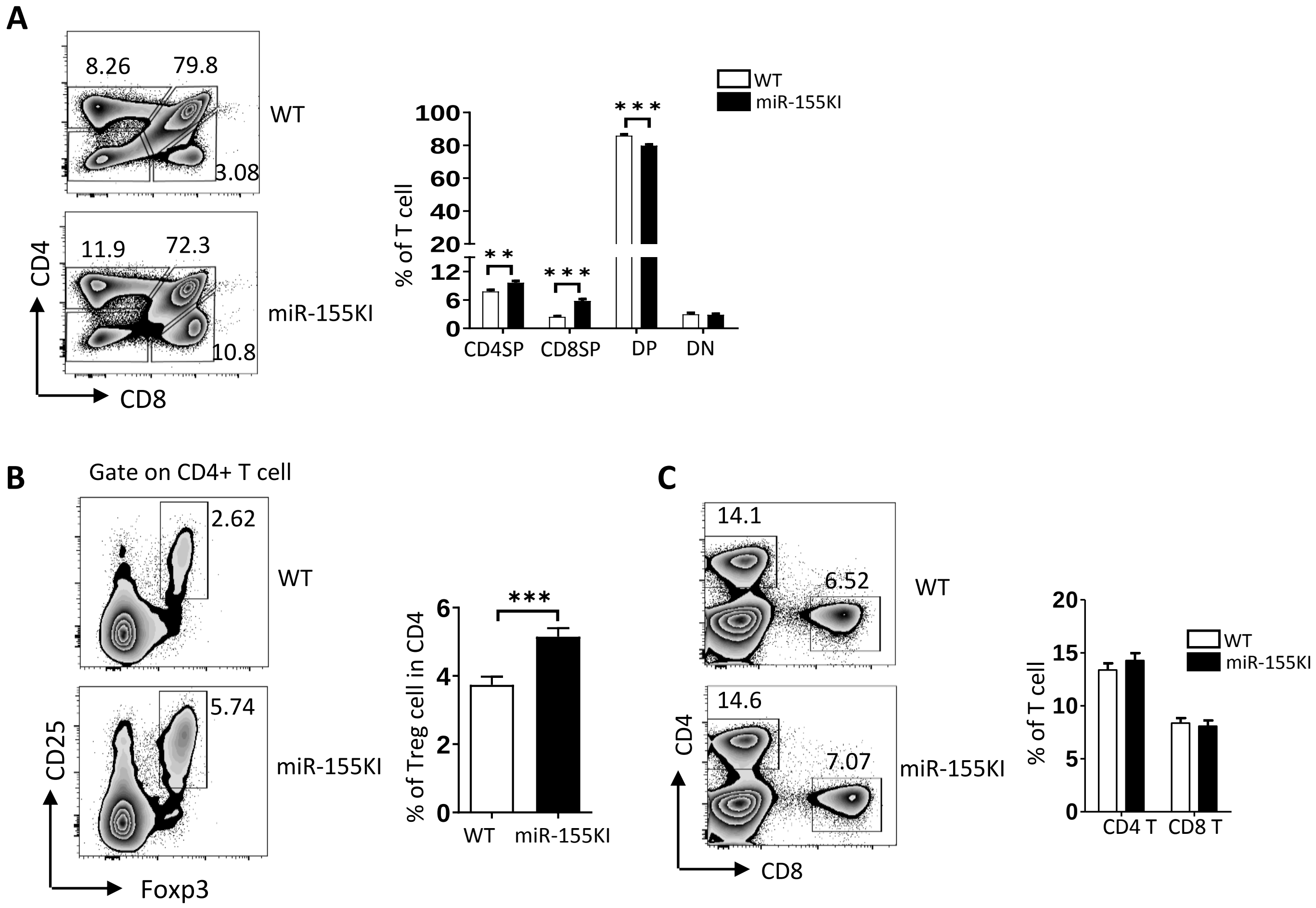

Supplemental Fig. 4 The role of miR-155 in conventional T cells

(**A**)Representative flow cytometric plots showing CD4$^+$ single positive (CD4 SP), CD8 SP, CD4$^+$CD8$^+$ double positive (DP) and CD4$^-$CD8$^-$ double negative (DN) in thymus from miR-155KI and WT controls (left); Bar graph showing the percentages of indicated populations in miR-155 KI and WT mice (right).

(**B**) Representative flow cytometric plots showing regular T cells (Tregs) gated on thymic CD4 SP cells (left). Bar graph showing frequency of Tregs in thymic CD4 SP cells.

(**C**) Flow cytometric plots showing CD4 T cells and CD8 T cells in spleen (left); Bar graph showing the percentages of indicated populations in miR-155 KI and WT controls (right). Data are from at least three independent experiments. Data were analyzed by unpaired t-test. $**P < 0.01$, $***P < 0.001$

Supplemental Fig. 5

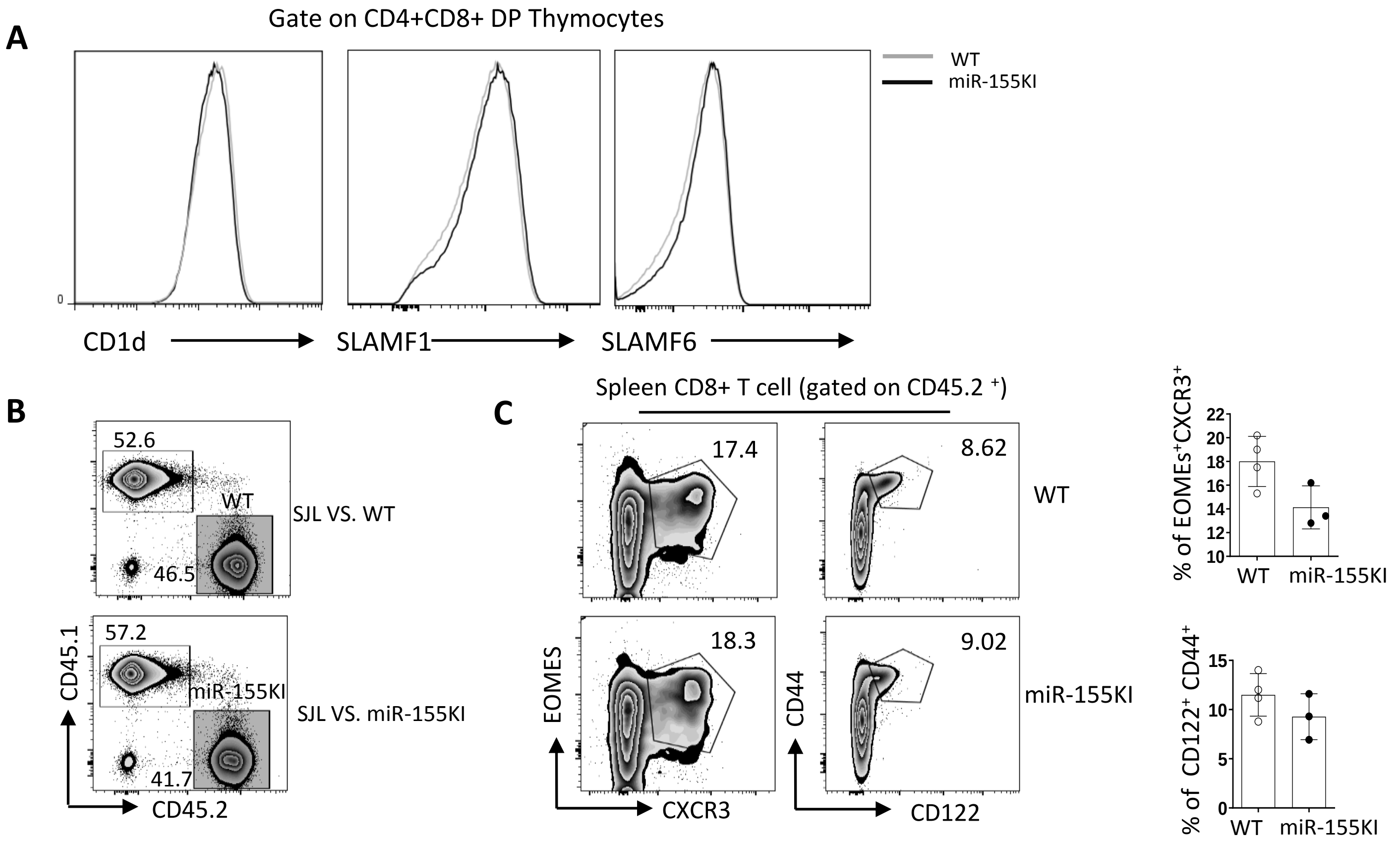

Supplemental Fig. 5 Cell-intrinsic versus extrinsic effects of defective *i*NKT cells and innate CD8 T cells with miR-155 overexpression

(A) Histogram showed CD1d, Slamf1 and Slamf6 expression in $CD4^{+}CD8^{+}$ double positive (DP) thymocytes from WT and miR-155KI mice. (B) Representative flow cytometry plots depicting thymic CD52.1 and CD45.2 expression. (C) Representative flow cytometry plots depicting splenic innate CD8 T cells (CXCR3+Eomes+ and CD44hiCD122+) (left);Bar graph showing frequency of splenic innate CD8 T cells derived from CD45.2+ WT and CD45.2+ miR-155KI BM (right).

Supplemental Fig. 6

A

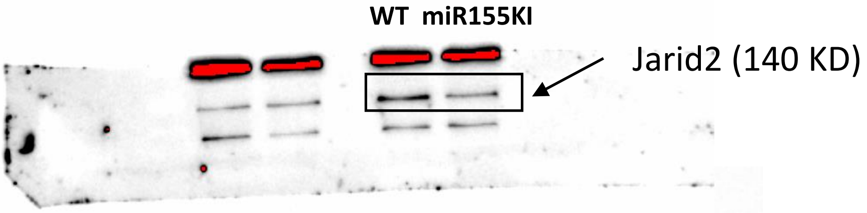

B

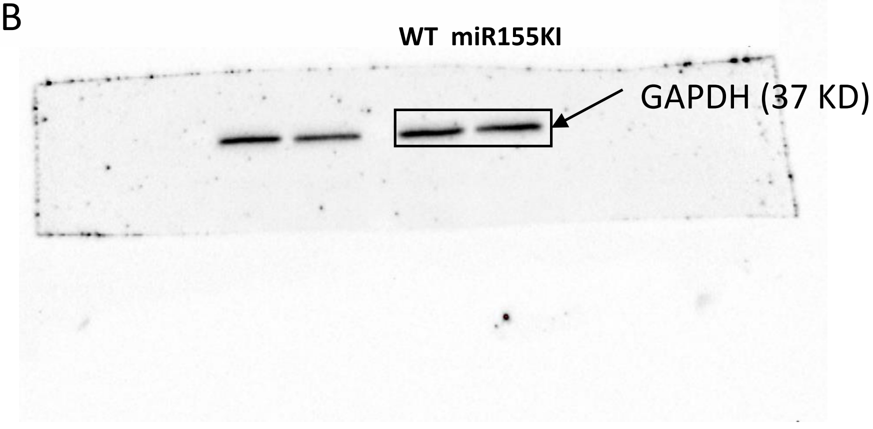

Supplemental Fig. 6 Full scans of the entire original blots for Fig. 8E

(A) Western blot analysis of Jarid2 protein (140KD, as indicated by the narrow) in total thymocytes from miR-155KI and WT mice. (B) Western blot analysis of GAPDH protein (37 KD, as indicated by the narrow) in total thymocytes from miR-155KI and WT mice from Figure A.

Supplemental Fig. 7

A NF-KAPPA B signaling pathway (miR-155KI vs. WT)

B TGF-BETA signaling pathway (miR-155KI vs. WT)

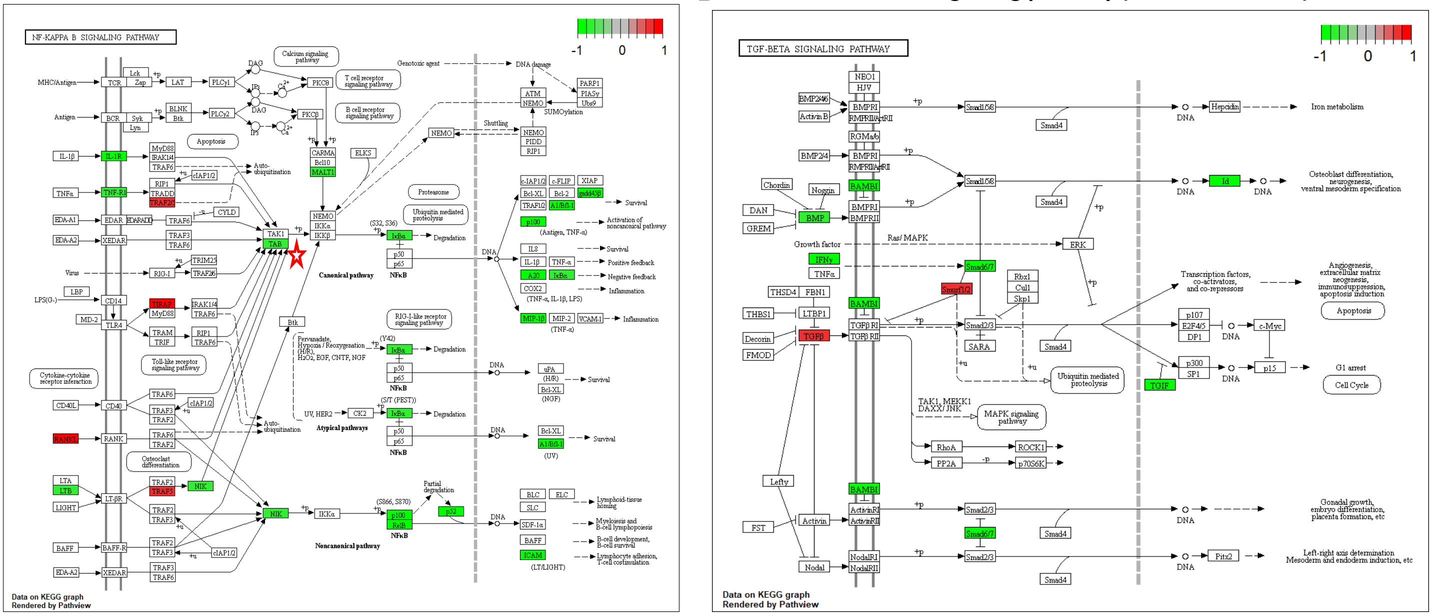

Supplemental Fig. 6 miR-155 overexpression interrupt NF-κB and TGF-β signaling pathway

(**A**,**B**) Key molecular dysregulation in NF-κB signaling pathway (A) and TGF-β signaling pathway (B) in *i*NKT cell developments (gene showing in green is down- regulated; gene showing in red is up-regulated, miR-155KI vs. WT).

Supplemental Table 1

PROBE	RANK IN GENE LIST	RANK METRIC SCORE	RUNNING ES	CORE ENRICHMENT
DDX3Y	60	1.595718026	0.06443853	No
ZSCAN12	220	0.81747967	0.017034916	No
ZBTB38	1024	-0.398365319	-0.4609354	Yes
RICTOR	1030	-0.403546065	-0.43826094	Yes
JARID2	1042	-0.409474075	-0.4189691	Yes
KRAS	1054	-0.415948302	-0.39926314	Yes
TRIM32	1065	-0.423640996	-0.37843826	Yes
KDM2A	1067	-0.426256001	-0.3518035	Yes
VPS18	1111	-0.464633763	-0.34904653	Yes
PLEKHA1	1120	-0.469108194	-0.3240598	Yes
CACUL1	1145	-0.492284447	-0.3076222	Yes
GLUL	1154	-0.499675184	-0.28068054	Yes
SARAF	1198	-0.544708431	-0.2728023	Yes
LRRC59	1210	-0.557816386	-0.24402301	Yes
WBP1L	1213	-0.56028676	-0.2094431	Yes
TAB2	1248	-0.611652613	-0.19164075	Yes
E2F2	1304	-0.684808493	-0.18232574	Yes
FOS	1306	-0.688597023	-0.13891263	Yes
MEF2A	1317	-0.709117889	-0.09982973	Yes
DHX40	1328	-0.722345889	-0.059900817	Yes
MAP3K14	1341	-0.749952674	-0.019460192	Yes
HBP1	1399	-0.876546025	8.64E-04	Yes
DYRK2	1421	-0.932848811	0.047359034	Yes
GABARAPL1	1481	-1.180885077	0.08589338	Yes

Supplemental Table 1: **Predicted miR-155 Target genes in *i*NKT cell with miR-155 overexpression.**
